# Effects of Alterations of Post-Mortem Delay and Other Tissue-Collection Variables on Metabolite Levels in Human and Rat Brain

**DOI:** 10.3390/metabo10110438

**Published:** 2020-10-29

**Authors:** Melissa Scholefield, Stephanie J. Church, Jingshu Xu, Andrew C. Robinson, Natalie J. Gardiner, Federico Roncaroli, Nigel M. Hooper, Richard D. Unwin, Garth J. S. Cooper

**Affiliations:** 1 Centre for Advanced Discovery & Experimental Therapeutics, Division of Cardiovascular Sciences, School of Medical Sciences, Faculty of Biology, Medicine and Health, The University of Manchester, Manchester Academic Health Science Centre, Manchester M19 9NT, UK; stephanie.church@manchester.ac.uk (S.J.C.); jingshu.xu@hotmail.com (J.X.); r.unwin@manchester.ac.uk (R.D.U.); garth.cooper@manchester.ac.uk (G.J.S.C.); 2 School of Biological Sciences, Faculty of Science, University of Auckland, Private Bag 92 019, Auckland 1142, New Zealand; 3 Division of Neuroscience & Experimental Psychology, School of Biological Sciences, Faculty of Biology, Medicine and Health, The University of Manchester, Salford Royal Hospital, Salford M6 8HD, UK; andrew.c.robinson@manchester.ac.uk; 4 Division of Diabetes, Endocrinology & Gastroenterology, School of Medical Sciences, Faculty of Biology, Medicine and Health, The University of Manchester, Manchester Academic Health Science Centre, Oxford Rd, Manchester M13 9PL, UK; natalie.gardiner@manchester.ac.uk; 5 Division of Neuroscience & Experimental Psychology, and Lydia Becker Institute of Immunology & Inflammation, School of Biological Sciences, Faculty of Biology, Medicine and Health, The University of Manchester, Manchester Academic Health Science Centre, Manchester M19 9NT, UK; federico.roncaroli@manchester.ac.uk; 6 Division of Neuroscience & Experimental Psychology, School of Biological Sciences, Faculty of Biology, Medicine and Health, The University of Manchester, Manchester Academic Health Science Centre, Manchester M19 9NT, UK; nigel.hooper@manchester.ac.uk; 7 Stoller Biomarker Discovery Centre & Division of Cancer Sciences, School of Medical Sciences, Faculty of Biology, Medicine and Health, The University of Manchester, Citylabs 1.0 (Third Floor), Nelson Street, Manchester M13 9NQ, UK

**Keywords:** Alzheimer’s disease, post-mortem delay, human brain metabolomics, rat brain metabolomics, mass spectrometry, brain tissue quality

## Abstract

The use of post-mortem human tissue is indispensable in studies investigating alterations in metabolite levels in neurodegenerative conditions such as Alzheimer’s disease (AD). However, variability between samples may have unknown effects on metabolite concentrations. The aim of this study was to characterize the impact of such variables. Cingulate gyrus was obtained from AD cases and controls, from three brain banks. Gas chromatography-mass spectrometry (GC-MS) was used to measure and compare the levels of 66 identifiable metabolites in these tissues to determine effects of tissue-collection variables. The effect of PMD was further investigated by analysis of rat brain cortex and cerebellum collected following post-mortem delays (PMDs) of zero to 72 h. Metabolite levels between cases and controls were not replicable across cohorts with variable age- and gender-matching, PMD, and control Braak staging. Analysis of rat tissues found significant effects of PMD on 31 of 63 identified metabolites over periods up to 72 h. PMD must be kept under 24 h for metabolomics analyses on brain tissues to yield replicable results. Tissues should also be well age- and gender-matched, and Braak stage in controls should be kept to a minimum in order to minimize the impact of these variables in influencing metabolite variability.

## 1. Introduction

Alzheimer’s disease (AD) is the most common form of age-related dementia and currently affects more than 44 million people worldwide. Its incidence is projected to more than triple by the year 2050 [1]. Despite AD being a leading cause of death in many countries, including the UK [2], there is still no treatment that can slow or halt the condition.

A lack of understanding of the pathogenesis of AD impacts the progress in developing effective treatments. Although research has aimed to determine the contributors to AD pathogenesis, including deposition of misfolded amyloid-β and tau, oxidative stress, inflammation, mitochondrial dysfunction, protein, and lipid dysregulation, such investigations have not yet led to the development of effective disease-modifying intervention [3]. Many of the identified perturbations involve metabolic processes such as purine catabolism, glucose metabolism, and amino acid pathways [4,5,6,7,8]. Such pathways may link seemingly disparate mechanisms and provide potential explanations for AD pathogenesis, and suggest new therapeutic targets. 

Currently, much research examining the role of these pathways in AD relies on pre-clinical models. However, human post-mortem brain tissue is vital for investigating the mechanisms of AD. Although post-mortem brains cannot represent a dynamic living system, tissues taken from individuals who died with AD can provide direct insight into the disease and can be compared to tissue from matched controls [9].

There are ‘known unknowns’ that apply to the use of human brain tissues for the investigation and modelling of AD in case-control studies. Factors that can vary either randomly or systematically among individual donors or institutions, include: cause of death, post-mortem delay (PMD), tau Braak stage [10], extent and severity amyloid deposits, vascular pathology and other co-existent pathologies, age, and gender. There is little information currently available on the impact of these factors on the metabolome—the composition of all metabolites present within a tissue—which, as a whole, has not yet been investigated.

This investigation aimed to determine the effects of identified variables on tissue-metabolite concentrations from human AD and control-brain tissue, with cingulate gyrus as the representative brain tissue. The effects of PMD, in particular, were further examined under controlled conditions by using healthy adult rat brain tissues.

## 2. Results

### 2.1. Cohort Comparisons in Human Brain Tissue

A comprehensive description and comparison of the human brain cohorts have been reported previously, with details for each individual sample [1]. In brief, the cingulate gyrus was obtained from nine AD cases and nine controls from each of three geographically distinct brain banks based in Auckland, New Zealand, Manchester, and Newcastle, UK. Cingulate gyrus tissue was selected as it is a region that is widely available from brain banks and has also been reported to show changes in several metabolites in AD [2]. It was also chosen as a more suitable pilot region for validation than more in-demand regions, such as the hippocampus. 

Tau Braak staging at time of death was, on average, found to be more advanced in Manchester (I-II; see Table 1) and Newcastle (I-II) controls than Auckland controls (0). Manchester controls (83 years) were older than Auckland controls (72 years) by around a decade on average. Both Newcastle cases (85 years) and controls (83 years) were on average older than Auckland samples by around a decade. Average PMD for the Manchester (82 h) and Newcastle (46 h) was also higher than Auckland samples (11 h).

Consortium to Establish a Registry for Alzheimer’s Disease (CERAD), SVD, Thal, and CAA scores were also acquired where available from brain bank databases (see Table 1 for CERAD and CAA cohort scores). Due to the limited availability of this data, this information could not be obtained for some samples in the UK cohorts, or for any of the Auckland samples. Thus, comparisons between cohorts were not possible. However, individual data are shown in Supplementary Materials A where available. Many cases from the Manchester and Auckland cohorts (three and fourteen respectively) had moderate to severe CAA. Five Manchester cases had moderate to severe SVD. Severity of CAA did not significantly differ between cases and in either cohort, nor did severity of SVD in the Manchester cases (data not shown). Where available, CERAD scores showed a significant correlation with Braak stage (*p* < 0.0001; see Supplementary Figure A1).

### 2.2. Human Brain Metabolite Analysis

Following a previous analysis on the Auckland cohort (Xu et al., 2016a), an investigation of two distinct cohorts from the Manchester and Newcastle brain banks was performed in order to investigate whether findings from different groups would produce similar findings. A total of 66 unique metabolites were identified in tissue from the cingulate gyrus of nine AD cases and nine controls in both the Manchester and Newcastle cohorts.

#### 2.2.1. Newcastle Cohort

**Figure 1:**
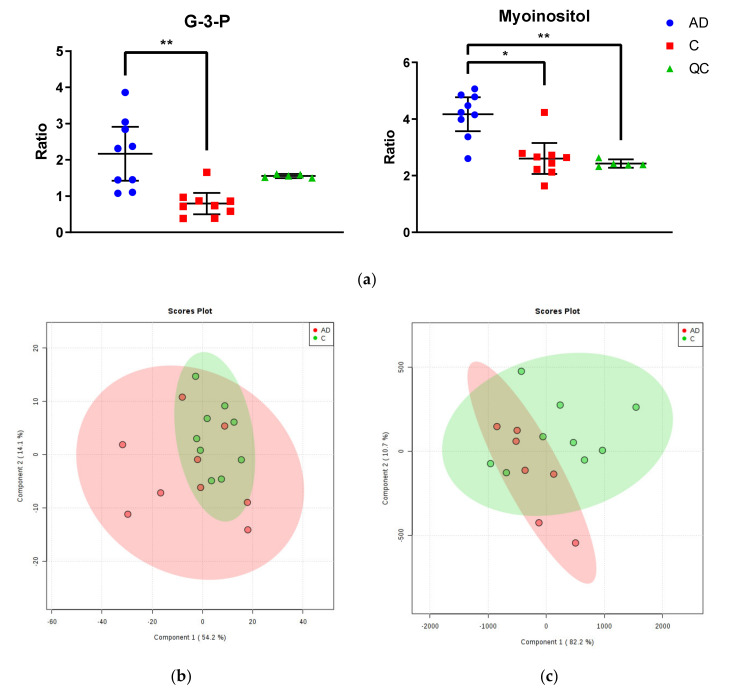
Metabolomic Analysis of Newcastle and Manchester Cohorts. (**a**) Glycerol-3-phosphate and myo-inositol were the only significantly altered metabolites between cases and controls in the Newcastle cohort. Graphs show ratios of significantly altered metabolites in comparison to internal standards within cingulate gyrus of cases (blue), controls (red) and quality control values (QCs; green). Error bars show ± 95% confidence intervals. Inter-group differences calculated by Kruskal–Wallis test: * *p* < 0.05; ** *p* < 0.01 (**b**) PLS-DA plot of metabolites in cingulate gyrus of the Newcastle cohort. Cases are shown in red and controls in green. See Supplementary Materials C for labelled and unlabelled PCA and PLS-DA plots. (**c**) PLS-DA plot of metabolites in cingulate gyrus of the Manchester cohort. Cases are shown in red and controls in green. See Supplementary Materials C for labelled and unlabelled PCA and PLS-DA plots.

Two significantly altered metabolites were identified within the Newcastle cohort: increased myo-inositol and glycerol-3-phosphate in cases compared to controls (see Figure 1a; see Supplementary Materials B for table of all identified metabolites). However, no separation was found between cases and controls, or between individuals of different gender, age, disease stage, PMD, CAA, or brain weight when mapped onto a (PLS-DA) plot (see Figure 1b). Both quality control measurements (QCs) and (QLs) were closely clustered (with the exception of the first QL; see Supplementary Figure C2), consistent with a successful GC run. 

#### 2.2.2. Manchester Cohort

No significantly altered metabolites were identified within the Manchester cohort (see Supplementary Materials B for table of all identified metabolites). Additionally, no separation was found between cases and controls, or between individuals of different gender, age, Braak staging, CERAD staging, CAA or SVD, PMD, or brain weight when mapped on to a PLS-DA plot (see Figure 1c). Again, QCs were tightly clustered, suggesting an accurate GC run. There were two outliers far removed from the other samples (cases AD5 ‘09/15′ and AD9 ‘10/17′; see Supplementary Materials C1.I.) within the case group, which were removed from the final analysis. These samples could not be distinguished from other individuals in the group by age, gender, disease staging, CAA/SVD, PMD, or brain weight.

#### 2.2.3. Comparison of All Cohorts

Although the results of the metabolite analysis within the Newcastle and Manchester cohorts were similar, they were markedly different from those previously found within the Auckland cohort [2]. Within the Auckland cohort, 28 of 69 identified metabolites were found to be significantly altered between the cingulate gyrus of cases and controls. One of these was glycerol-3-phosphate, which was 1.8-fold higher in cases than in controls; the only finding that was also present in the Newcastle cohort. However, unlike the Newcastle cohort, no significant change was found in myo-inositol between Auckland cases and controls. None of the case-control differences present in the Auckland group were found in the Manchester cohort. Significant findings in the Auckland group included large (5-fold or more) increases of several metabolites in cases compared to controls, including glucose, sorbitol, fructose, glucose-6-phosphate, and fructose-6-phosphate, all involved in glucose metabolism pathways [2]. There were no significant changes in any of these metabolites found in either of the UK cohorts.

### 2.3. Effect of PMD on Metabolomic Analysis of Rat Brain Tissue

In order to determine any effects of PMD on metabolite levels within the brain in a more controlled manner, a metabolomics analysis was carried out on the brains of rats controlled for both age and gender, with PMDs of 0, 24, 48, and 72 h. 63 unique metabolites were measured in the post-mortem cerebral cortex and cerebellum of forty healthy adult Wistar Han rats.

**Figure 2:**
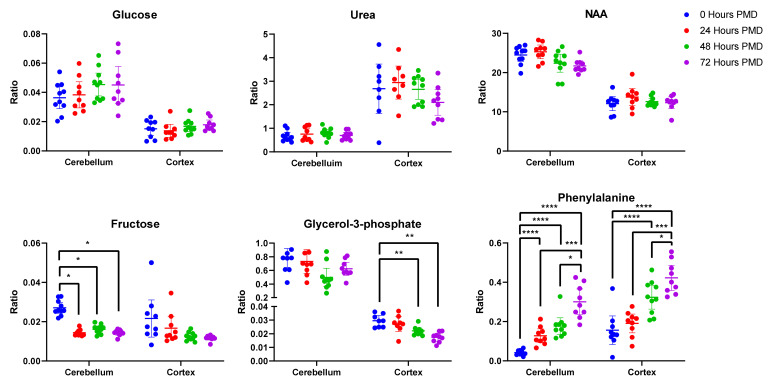
Unaltered and Altered Metabolites in Rat Brain Tissue. Data shown is individual metabolite ratios ± 95% confidence intervals. 23 identified unique metabolites were unchanged in either the rat cortex or cerebellum over a period of 72 h post-mortem delay (PMD), including metabolites previously associated with AD such as glucose, urea, and n-acetylaspartic acid. In the cortex, 22 metabolites were altered by 72 h PMD including glycerol-3-phosphate and phenylalanine. 23 metabolites were altered in the cerebellum by 72 h PMD, including fructose and phenylalanine. * < 0.05, ** < 0.01, *** < 0.001, **** < 0.0001. PMD = post-mortem delay. Individual sample values, mean ratios, and fold-changes for all metabolites in the cortex and cerebellum can be found in Supplementary Materials D.

Of these metabolites, 21 were unchanged in either region over the whole period of up to 72 h PMD (see Table 2). Of these, four showed a coefficient of variation (CV) higher than 30% in the quality controls (QCs) in at least one of the brain regions and so were discarded (alanine, tryptophan, glutamine, hexanedioic acid). Phthalic acid was unchanged in the cortex, but was not identified in the cerebellum (see Figure 2). Included in the 16 remaining metabolites were glucose, urea, lactic acid, and several carboxylic and fatty acids such as benzoic acid, malic acid, and phosphoric acid. Among the unaltered metabolites were molecules that have been previously associated with AD, such as glucose, urea, and n-acetylaspartic acid (NAA; see Figure 2).

Of 57 unique metabolites identified in the cortex, four showed a CV higher than 30% in the QCs and so were discarded (proline, hypoxanthine, tryptophan, and pyruvic acid). Of the remaining 53 metabolites, 22 were found to be significantly different between 0 and 72 h PMD. Around 75% of the altered metabolites increased in concentration from 0 to 72 h, most markedly cysteine (7.2-fold change; see Table 3), propanoic acid (5.8-fold change), and glycine (4.1-fold change). Others decreased, most notably adenosine (50-fold change). Of the altered metabolites in the cortex, ten already showed statically significant changes by 24 h (see Supplementary Materials D). Some showed continuous changes across the entire 72-h period, such as glycine, phenylalanine (see Figure 2), and cysteine. Others stopped increasing or decreasing after 48 h, including adenosine and glycerol-3-phosphate (see Figure 2). As such, 24 h PMD was sufficient to induce changes in several metabolites, with some changes peaking at 48 h, and others continuing to change up to 72 h and possibly beyond. 

**Figure 3:**
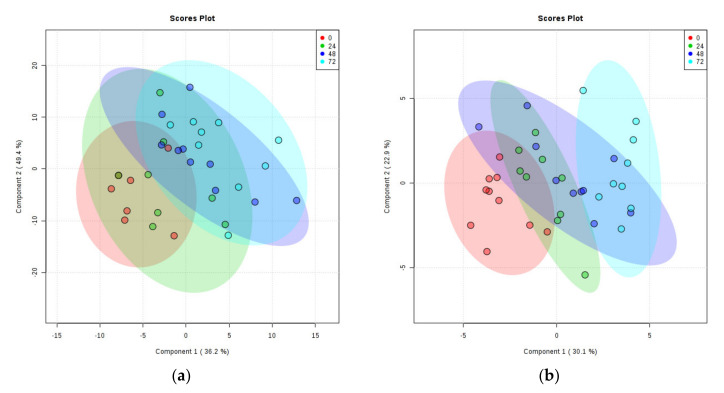
PLS-DA Plots of Rat Brain Tissue Metabolites. (**a**) Rat cortex. (**b**) Rat cerebellum. Ratios of identified metabolites in comparison to selected internal standards within rat brain tissues at 0-h PMD (red), 24 h PMD (green), 48 h PMD (blue), and 72 h PMD (purple). A shows cortex; B shows cerebellum. See Supplementary Materials E for labelled and unlabelled PCA and PLS-DA plots.

There was a clear separation of PMD groups on the PLS-DA plot of rat cortex metabolites, with the 0, 24, 48, and 72 h separating linearly in numeric order (see Figure 3a). Three samples and one quality lead (QL) were identified as outliers and removed from the plot (see Supplementary Materials E).

Fifty-four unique metabolites were identified in the rat cerebellar tissues. Of these, seven showed a CV higher than 30% in QCs (alanine, hexanedioic acid, glutamine, hypoxanthine, tyrosine, 9H-purin-6-amine, and ascorbic acid) and were excluded. Of the remaining metabolites, two-way ANOVA showed that 23 differed significantly across different time points. These all showed increases in the 72-h PMD group compared to the 0-h PMD group, with the exception of adenosine minor, which decreased ten-fold, and fructose, which showed a two-fold decrease (see Table 3 & Figure 2). The largest increases were seen in methionine (7.5-fold), and phenylalanine (9.0-fold; see Figure 2). All but two of the altered metabolites in the cerebellum (threonine and uridine) already showed significant changes by 24 h PMD (see Supplementary Materials D).

PLS-DA plotting again showed clear separation of different PMD groups, sequentially from 0 to 72 h (see Figure 3b). One sample was identified as an outlier and removed from the plot (see Supplementary Materials E for labelled data points).

## 3. Discussion

### 3.1. Metabolomic Analysis

This study shows that measured levels of metabolites appear to be highly sensitive to variations in PMD. This finding follows a recent case-control study we conducted to investigate the effects of PMD, Braak stage, age, and brain bank location using the Auckland and Manchester cohorts. The study found no effect of any of these variables on levels of essential metals in brain tissue [1]. As such, it appears that brain metabolites do not show the same robustness to variability as metals do. No significant case-control differences in metabolite levels were observed in the Manchester cohort and only two metabolites were significantly different between AD cases and controls in the Newcastle group, in comparison to almost thirty metabolites within the Auckland cohort. Tight clustering of QCs in both UK cohorts suggests no errors with the running of the GC analysis, and that the given ratio values are precise. Additionally, repeats of two Auckland cases and controls that had been stored at −80 °C for two years were analyzed alongside the Manchester cohort and showed the same PLS-DA plot pattern as in the original analysis, consistent with reproducibility of results even when the same samples were analyzed several years apart (data not shown).

As such, it appears that the lack of case-control differences observed within the groups may be due to the characteristics of the groups themselves. The Newcastle cohort was age, PMD, and gender-matched, whilst the Manchester cohort was not matched by any of these variables. Despite this, neither showed any of the case-control differences in metabolite levels observed in the Auckland cohort. This suggests that the lack of case-control differences observed in the two UK cohorts are not accounted for by case-control matching by these factors. However, both cohorts had cases and controls with higher tau Braak stage, as well as higher control ages, than the Auckland group. It is possible that the marked differences observed between the UK and Auckland cohorts could be influenced by either or both of these variables. Although there are many studies on metabolite levels in AD that go on to investigate correlations with Braak stage, few studies could be found measuring metabolite levels at individual stages [3] and comparing them directly, and no such reports could be found that directly compared levels at Braak stage 0, I and II. As such, it is difficult to tell what the effect of Braak staging may be on metabolite levels in our own investigations, or whether differences in low Braak stages between controls in different cohorts may be exerting an effect on the metabolomic analyses. 

Investigations on the effects of aging on selected metabolites in healthy brains have indicated that some metabolites are sensitive to increases in age, with reports of decreased NAA and increased myo-inositol in the brains of older healthy individuals [4]. These changes are usually reported in comparisons of young (<50 years) and older (>50 years) groups, but can also be seen in investigations including only older individuals [5,6,7]. However, to date, there have been no studies on the effects of aging on the brain metabolome as a whole, with studies generally restricted to metabolites, which can be imaged in living brains using MRI. This makes it difficult to assess the exact influence of control age on the findings reported here, but in combination with the present findings indicates that increased age most likely has an effect on at least some of the metabolites reported here, such as myo-inositol, even in healthy individuals. 

To the author’s knowledge, there have to date been no investigations on the effects of antemortem events on brain metabolite levels, although a study by Durrenberger and colleagues has observed that events such as hospitalization, coma, and ventilation prior to death are associated with lower RNA quality across six different regions of the human brain including the cerebellum [8]. Several such antemortem events may be indicated by the causes of death recorded for the samples used in this study, but due to the large variations in and numbers of such events, as well as a lack of cause-of-death data for many samples, it is difficult to directly investigate any effects they may have here. However, the study by Durrenberger et al. also found no effect of PMD on brain RNA quality, a finding that has been replicated elsewhere [9,10,11]. As such, these observations on the effects of antemortem effects on RNA may not be reflected in studies looking at metabolites. A previous study looking at the effects of PMD on brain metabolite levels has reported changes in the hippocampi of healthy mice as early as two hours post-mortem, despite a lack of anatomical changes in neurons and glia for up to five hours [12]. Another study comparing changes in nucleoside levels between rat and human brains at varying levels of PMD observed several significant changes from two hours onwards, with similar patterns of change but differing concentrations between species [13]. What is particularly lacking in the current literature is an untargeted metabolomics study comparing the effect of PMD across different brain regions within the same brain for delays of longer than 24 h. Considering the length of PMD commonly found with human brain samples, studies of such longer periods are required to understand the effect of this variable on human brain tissue experiments. However, this is difficult to perform in human samples, as the same brain cannot be used to look at different PMDs due to the nature of sample collection, and there are too many variables involved in comparing samples from multiple donors. This type of experiment is, however, feasible using animal models, which can also be easily controlled for age, gender, and other factors.

### 3.2. Effect of PMD on Metabolomic Analyses in Rat Brain Tissues

In this study, healthy adult rat tissues were used to look at the effect of different PMDs on cerebral metabolites; a parameter that varied greatly between different brain banks and individual human samples, whilst tightly controlling for the effects of age and gender.

The literature on the effect of PMD on rat brain tissues is limited. One study found a steady decrease in catecholamine concentration in multiple rat brain regions (including the cortex and cerebellum) up to a period of 54 h post-mortem [14]. Another study reported changes in proteins such as syntaxin in both human and rat cortex samples by 48 h [15]. Importantly, there were also decreases in post-synaptic density protein 95 (PSD-95) noted in human samples by 24 h, whereas rat cortex PSD-95 remained stable for the entire measured period of 72 h [15]. This highlights the limitations that need to be kept in mind when applying animal data to human samples. Protein changes have also been observed to occur within the caudate-putamen, hippocampus, and medulla of rat brains in a period of up to 72 h [16]. These changes were not consistent across different areas of the brain, showing regional differences in susceptibility of the rat brain to such alterations, at least for protein concentrations.

A 2005 study by Kovacs and colleagues found that levels of several nucleosides were significantly altered by 24 h PMD, and some even by as little as 2 h in rat cortex samples [13]. In our experiment, inosine showed no significant changes in the cortex, contrary to decreases reported by Kovacs and colleagues. However, both Kovacs et al. and the present study observed a significant decrease in adenosine and increase in uracil concentrations by 24 h PMD. Additionally, Kovacs et al. reported no changes in uridine over 24 h; this was also observed here, with no decrease in uridine concentration until 48 h. As such, several observations from the rat cortex were replicated here. However, this study was unable to detect all the nucleosides measured by Kovacs et al. (such as xanthine and hypoxanthine). It should also be noted that concurrent experiments run on human samples by Kovacs and colleagues showed similar patterns in nucleoside changes in human brain samples, although overall concentrations differed. It is unknown what effect other variables in the human tissues such as age, gender, or cause of death may have had on these results; however, this does demonstrate an applicability of observations in rat brains to human brains, although still with limitations.

Of 56 unique metabolites identified in our rat tissue samples, 23 showed no change in either the cortex or the cerebellum up to 72-h PMD (see Table 2). Among these unaltered metabolites were molecules that have been previously associated with AD. For example, both glucose and urea were seen both in the Auckland cohort reported here [2], with increased glucose also observed in other reports [17]. Both were unaffected by PMD in rat brains here (see Figure 2). However, unlike the Auckland cohort, glucose also showed no case-control differences in the Manchester and Newcastle human cohorts. This could be due to several other factors that differed between these groups, such as control age, disease staging, or cerebral vascular pathology. Alterations in glucose metabolism have been observed even in healthy aged brains [18], and glucose hypometabolism has been reported in preclinical AD cases with mild cognitive impairment and low Braak stage [19]. As such, the higher age or Braak stage of UK controls could contribute to the lack of case-control changes seen in the Manchester and Newcastle cohorts. Increased measures of cerebral vascular pathology such as CAA have also been associated with higher tau Braak stage AD, independently of other variables including CERAD, age, and gender [20]. This correlation between CAA score and Braak stage has also been observed in a separate cohort of samples with Braak stage 0-III pathology, with individuals more likely to be classified as Braak stage III when they showed CAA pathology [21]. Significant interactions between CAA status and measures of cognition and memory were also reported in this cohort, suggesting that CAA pathology may have contributed to cognitive impairment in these cases. However, there were no significant case-control differences in severity of CAA in either the UK cohorts, nor severity of SVD in the Manchester samples (see Table 1 & Supplementary Materials A). Differences in the latter could not be determined in the Newcastle cohort due to a lack of information available for samples. Likewise, neither CAA nor SVD scores were available for the Auckland samples, so it is unknown if case-control differences in either of these factors contributed to case-control separation.

NAA has been reported as decreased in AD brains [4,22,23], but likewise appeared unaffected by PMD in rat brains (see Figure 2). Investigations of these molecules should be robust in cohorts with PMDs of up to 72 h or with case-control differences in PMD. However, 22 metabolites did show significant changes in the cortex over a 72-h period, as well as 23 in the cerebellum (see Table 3). Many of these metabolites had already begun to show changes by 24 h and all by 48 h, similar to previous studies. This has significant implications for the use of brain tissues in metabolomics experiments, both human and animal. Although it cannot be assumed that human brain tissues will show all the same changes as those observed here in rat tissues, our data, in combination with previous reports, suggest that the human brain metabolome will have already undergone significant alterations in a time frame as short as 24 h. As such, it becomes imperative to limit post-mortem delay in these tissues as much as possible—at least within a 24-h period, but possibly in an even shorter time frame. Further studies looking at concentrations of the metabolites shown here to be affected by 24 h would be necessary to determine the maximum PMD allowable for reliable determination of these analytes. Although 14 of the metabolites observed to significantly change were common to both regions, seven were only altered in the cortex and nine in the cerebellum. This also raises the importance of recognising differences in susceptibility across brain regions and how the metabolome may be affected in each. Taken together, these data suggest that although targeted analyses of certain metabolites can be performed regardless of PMD of tissues, such as glucose and urea, untargeted analyses investigating the metabolome as a whole require careful control of PMD to at least under 24 h.

### 3.3. Conclusions

Taken together, the results shown here from both human and rat tissues illustrate a marked effect of PMD and a probable effect of disease stage and age on the brain metabolome. In particular, several metabolites showed alterations by PMDs of 24 h or more, which were not always consistent across multiple regions—making the matching of brain regions and precise dissection of tissues important if findings between patients and case-control groups are not to be ambiguous. As such, in order to maximize interpretability of metabolomics data in human brain tissue, it can be recommended that tissues should have a PMD of less than 24 h, although even this period may be too long for reliable measurements of many metabolites. Although the exact effects of age and Braak staging are difficult to determine, it seems likely that they also contribute to the differences in findings observed between human cohorts reported here. As such, samples should also be as well-matched in these areas as possible, and consideration should be made as to whether it is suitable to include low Braak stage (Braak stage I-II) individuals in AD control cohorts.

## 4. Materials and Methods 

The cohorts used in this study have been extensively characterized in a previous metallomics study [1]. In brief, human cingulate gyrus samples were obtained from 9 AD cases and 9 normal controls without neurological conditions, from each of three geographically-distinct brain banks: the Manchester Brain Bank based at Salford, UK; the Newcastle Brain Bank based at Newcastle, UK; and the New Zealand National Brain Bank based at Auckland, New Zealand. Patient metadata were obtained for variables including the following: age at death, gender, tau Braak stage, CERAD, Thal phase, small vessel disease (SVD) score, cerebral amyloid angiopathy (CAA) score, and PMD for each donor. 

Cortical and cerebellar tissues from twenty healthy adult Wister Han rats were also investigated for this study using a previously described protocol [1]. Rats were terminally anaesthetized (isoflurane), culled by decapitation, and stored at 4 °C for post-mortem intervals of 0, 24, 48, and 72 h before collection of brain tissues.

### 4.1. Diagnosis & Severity of Human Cases

All cases and controls were diagnosed at post-mortem examination by consultant neuropathologists at the respective brain bank. All cases had a confirmed diagnosis of AD, whereas controls only showed age-related changes. Characteristics of individuals and cohorts are included in Supplementary Table A.

### 4.2. Tissue Dissection

Brain tissues were dissected into samples of 50 mg (±5 mg) for GC-MS (gas chromatography/mass spectrometry) analysis using a metal-free ceramic scalpel. Samples were placed in ‘Safe-Lok’ microfuge tubes (Eppendorf AG; Hamburg, Germany) and stored at −80 °C until analysis.

### 4.3. GC-MS

GC-MS metabolites analysis was performed as previously described [2,24,25]. Samples from each brain bank were analyzed in separate runs. In brief, brain tissue was extracted in 50:50 (*v*/*v*) methanol:chloroform containing labelled internal standards (Citric acid-*d*_4_, ^13^C_6_-d-fructose, l-tryptophan-*d*_5_, l-alanine-*d*_7_, stearic acid-*d*_35_, benzoic acid-*d*_5_, and leucine-*d*_10_). Tubes containing the methanol:chloroform solvent (but not sample) were prepared for extraction blanks. Samples were lysed in a TissueLyser batch bead homogenizer (Qiagen, Manchester, UK) before addition of water and separation of polar and non-polar phases by centrifugation. The polar phase was transferred to a new tube and dried overnight in a Speedvac centrifugal concentrator (Savant Speedvac, Thermo Scientific, UK). An additional 200 µL was taken from each sample and pooled in order to create a quality control (QC), which was used to monitor any variation in GC readings over the course of the run. Quality control leads (QLs) were quality controls included at the beginning of the run as lead-ins in order to avoid variation in GC readings of samples during start-up. Samples were stored overnight at 4 °C before derivatization. Methlyoxime/trimethysilyl (TMS) derivatization was used, and retention markers added to each sample before centrifugation. Samples were taken immediately for GC-MS analysis.

GC-MS analysis was performed using an Agilent/J&W DB17-MS column (30 m × 0.25 mm × 0.25 μm) with a 3 m × 0.25 mm retention gap, and helium carrier at a constant flow of 1.4 mL/min. Ten spectra per second were acquired over the mass range of 45–800 Da in order to detect TMS derivatives of amino acids, sugars, sugar alcohols, organic acids, and miscellaneous organic molecules. QL samples were placed as a lead-in before and QCs were interleaved evenly between samples. Extraction blanks were included at the beginning and towards the end of the run to confirm the absence of carryover in each run. Samples were randomized in each run. 

### 4.4. Data Acquisition and Reduction

Data were prepared by a ‘Reference Compare’ method in ChromaTOF 4.5 (LECO; UK), using the NIST Mass Spectral Reference Library (NIST08/2008; National Institute of Standards and Technology/Environmental Protection Agency/National Institutes of Health Spectral 262 Library; NIST, Gaithersburg, MD, USA); and the Golm Metabolome Database (Max Planck Institute of Molecular Plant Physiology, Potsdam-Golm, Germany). Chromatographic retention-time data were obtained through our own in-house library reference standards. Identified metabolites were classified according to the Metabolomics Standards Initiative (MSI) minimum reporting standards criteria [26], i.e., (1) Confidently identified compounds (good match to library spectra and retention index); (2) Putatively identified compounds (matching of mass spectra only); or (3) Putatively characterized compound classes (matching of mass spectra only to chemical class of compound).

Expected retention time (based on the library retention times) and peak shape were manually integrated to constitute a definitive molecular identification, using identified metabolites from QC pooled samples as a representative reference list for the human cingulate gyrus. After removal of ambiguous and low-quality spectra, a total of 83 unique signals were identified and selected from the GC-MS chromatogram for use in the reference list. This list was then applied to every sample, using a tolerable retention-time deviation of 6 s and manual validation to ensure correct peak integration. Instead of raw peak areas, internal standard ratios were used for each metabolite; this was achieved by determining the heavy internal standard that yielded the lowest variance for each individual metabolite across all the QC injections.

### 4.5. Data Analysis

Multivariate principal-component analysis (PCA) and partial least squares discriminant analysis (PLS-DA) were performed on the entire list of identified metabolites in order to identify any separation of the samples into groups by applying MetaboAnalyst software (metaboanalyst.ca; Canada). Samples located far away from the sample clusters on plots were identified as outliers and removed from the final analysis (See Supplementary Materials C & E for plots both with and without outliers). Statistical significance of individual metabolites was calculated using multiple 2-tailed t-tests corrected for potential effects of multiple comparisons by applying a false-discovery rate (FDR) of 10% using GraphPad v6.04 (Prism; La Jolla, CA, USA). Metabolites with a coefficient of variation (CV) ≥30% in quality controls were disregarded. Where multiple signals were identified for a single metabolite, the adduct with the strongest signal was used in this analysis. However, a list of all identified signals is included in Supplementary Materials B & D.

## Figures and Tables

**Table 1:** Comparison of Brain Bank Groups.

Variable	Manchester Controls	Newcastle Controls	Auckland Controls
**Number**	9	9	9
**Age**	89 (82–95)	85 (76–94)	73 (61–78) ^a,c^
**Male Gender, *n* (%)**	6 (66.7)	6 (66.7%)	7 (53.8)
**Braak Stage**	I-II (0-I–II)	I-II (I–II)	0 (0–II) ^a,c^
**PMD (hours)**	75 (49–130)	25 (9–40) ^b^	12 (5.5–15.0) ^c^
**Whole Brain Weight (g) †**	1160 (1020–1494)	1235 (1064–1406)	1260 (1094–1461)
**CAA Score**	None/Mild	Moderate *	NA
**CERAD**	0 (0–A)	0 (0)	NA
**Variable**	**Manchester Cases**	**Newcastle Cases**	**Auckland Cases**
**Number**	9	9	9
**Age**	83 (61–89)	83 (70–95)	72 (60–80) ^a^
**Male Gender, *n* (%)**	3 (33.3)	6 (66.7%)	5 (55.6)
**Braak Stage**	IV-V (IV–VI)	VI (VI–VI) ^b^	V-VI (IV–VI) ^c^
**PMD (hours)**	39 (12–70)	25 (9–41) ^b^	7 (4.0–12.0) ^c^
**Whole Brain Weight (g) †**	1066 (900–1359)	1155 (959–1351)	1062 (831–1355)
**CAA Score**	Mild/Moderate	Moderate/Severe *	NA
**CERAD**	C (B–C)	C (B–C) *	NA

Age, male (%), Braak stage, PMD are means (range); CERAD scores are modes (range); CAA scores are modes; *p*-values for significance of between-group differences were calculated by one-way ANOVA followed by Tukey’s test. ^a^ *p* < 0.05 between Newcastle and Auckland cohorts; ^b^ *p* < 0.05 between Newcastle and Manchester cohorts; ^c^ *p* < 0.05 between Manchester and Auckland cohorts. See Supplementary Materials A for details of individual cases and controls. CAA = Cerebral amyloid angiopathy; PMD = post-mortem delay; NA = Not available. * Data not available for some samples (see Supplementary Materials A); † some brains weighed at point of fixation by brain bank (see Supplementary Materials A)

**Table 2:** Unaltered Metabolites in Rat Brain Tissue.

Metabolite	Cortex					Cerebellum				
	Ratio at 0 h	Ratio at 24 h	Ratio at 48 h	Ratio at 72 h	*p*-Value (0 vs. 72 h)	Ratio at 0 h	Ratio at 24 h	Ratio at 48 h	Ratio at 72 h	*p*-Value (0 vs. 72 h)
***Alternative Fuel Sources***										
**Lactic acid ^2^**	0.6	0.5	0.6	0.6	0.9	0.4	0.5	0.5	0.5	0.8
***Amino Acids and Related Compounds***										
**Hydroxylamine ^2^**	3.0	3.7	1.6	1.8	0.8	1.9	1.4	1.5	1.2	0.5
**N-acetylaspartic acid (NAA)^1^**	12.3	13.8	12.6	12.3	0.9	24.5	25.3	22.4	21.7	1.0
**Pyroglutamic acid ^1^**	0.02	0.02	0.02	0.02	1.0	0.002	0.002	0.002	0.002	0.8
***Glucose & Related Metabolites***										
**Glucose ^2^**	0.01	0.01	0.02	0.02	0.3	0.04	0.04	0.05	0.05	0.8
***Other***										
**1-Methyl-N,N-bis(trimethylsilyl)-4-[(trimethylsilyl)oxy]-1H-imidazol-2-amine ^2^**	41.7	42.6	45.1	47.2	0.6	2.5	2.9	2.6	2.2	0.9
**1-piperidinecarboxaldehyde ^2^**	0.05	0.07	0.03	0.03	0.4	0.3	0.3	0.3	0.3	0.9
**7,9-Di-tert-butyl-1-oxaspiro(4,5)deca-6,9-diene-2,8-dione ^2^**	0.01	0.02	0.01	0.01	0.4	0.1	0.2	0.2	0.2	1.0
**Benzoic acid ^2^**	0.03	0.03	0.02	0.02	0.3	0.008	0.007	0.009	0.009	0.6
**Malic acid ^2^**	0.6	0.7	0.7	0.7	0.4	0.06	0.06	0.07	0.07	0.7
**Palmitic acid ^1^**	5.4	8.0	6.4	6.7	0.3	4.2	4.8	4.8	5.8	0.2
**Phthalic acid ^2^**	3.3	4.1	2.8	3.0	1.0	NI	NI	NI	NI	NI
**Phosphoric acid ^2^**	0.2	0.1	0.0	0.0	0.07	0.05	0.04	0.05	0.04	0.5
**Silanol ^2^**	15.2	17.0	14.9	14.8	1.0	13.2	14.4	13.0	14.0	1.0
**Stearic acid ^1^**	2.6	4.4	2.9	3.0	0.4	5.9	6.7	6.7	8.1	0.2
**Threitol ^2^**	0.07	0.09	0.09	0.09	0.2	0.007	0.009	0.010	0.010	0.7
**Urea ^2^**	5.7	2.6	2.7	2.1	0.09	0.7	0.8	0.8	0.7	0.7

Table shows mean ratio values (full list of values can be found in Supplementary Materials D). *p*-value between 0 and 72 h PMD determined by two-way ANOVA. NI = Not identified. ^1^ Confidently identified compounds (good match to NIST and in-house library spectra and retention index); ^2^ Putatively identified compounds (matching of mass spectra only).

**Table 3:** Metabolites Significantly Altered in Rat Brain Tissue.

Metabolite	Fold-Change	
	Cortex	Cerebellum
***Alternative Fuel Sources***		
**Glycerol-3-phosphate ^1^**	**0.5**	0.8
***Amino Acids and Related Compounds***		
**Aspartic acid ^1^**	1.2	**2.3**
**Cysteine^1^**	**7.2**	**5.9**
**Ethanolamine ^1^**	0.7	1.9
**GABA ^2^**	1.0	**2.0**
**Glycine ^1^**	**4.1**	**2.1**
**Isoleucine ^1^**	**2.1**	**3.8**
**Leucine ^1^**	**2.2**	**3.2**
**Methionine ^1^**	1.3	**6.4**
**Phenylalanine ^1^**	**3.2**	**9.0**
**Proline ^1^**	1.5	**7.2**
**Serine ^2^**	1.3	**2.3**
**Threonine ^1^**	0.5	**1.8**
**Tyrosine ^1^**	**3.8**	NI
**Valine ^1^**	**2.1**	**3.4**
***Glucose & Related Metabolites***		
**Fructose ^1^**	0.7	**0.5**
**Ribose ^2^**	**2.0**	**2.5**
**Ribose-5-phosphate ^1^**	1.6	**2.4**
**Sorbitol ^1^**	**1.7**	**1.7**
***Nucleosides***		
**Adenosine ^1^**	**0.05**	**0.1**
**Uracil ^2^**	**2.8**	**3.2**
**Uridine ^1^**	**0.3**	**0.3**
***Other***		
**9H-purin-6-amine ^2^**	**1.8**	EXC
**Hydroxybutyric acid ^1^**	1.3	**2.0**
**Pantothenic acid ^1^**	**1.9**	1.4
**Butanedioic acid ^2^**	**2.0**	**2.4**
**Ethylbis(trimethylsilyl)amine ^2^**	**0.2**	1.0
**Ascorbic acid ^2^**	**0.2**	EXC
**Propanoic acid ^2^**	**5.8**	**5.0**
**Pyroglutamic acid ^1^**	**0.3**	0.9
**Glycerol ^2^**	**3.1**	**4.4**

Significantly altered (*p* < 0.05) metabolites between rat brain tissue PMD groups in each region are highlighted in bold. Mean fold-changes are between 0 and 72-h PMD groups. NI = not identified; EXC = excluded due to QC CoV ≥ 30%. ^1^ Confidently identified compounds (good match to NIST and in-house library spectra and retention index); ^2^ Putatively identified compounds (matching of mass spectra only).

## Supplementary Materials

The following are available online at https://www.mdpi.com/2218-1989/10/11/438/s1, Table A1: Characteristics of individuals in the Manchester cohort, Table A2: Characteristics of individuals in the Newcastle cohort, Table A3: Characteristics of individuals in the Auckland cohort, Figure A1: CERAD Score vs tau Braak Stage, Figure C1. Manchester Cohort PCAs and PLS-Das, Figure C2: Newcastle Cohort PCAs and PLS-Das, Supplementary Material C: Human Brain Plots, Supplementary Material D: Rat Data; Supplementary Figure E1. Rat Cortex PCAs and PLS-Das, Figure E2. Rat Cerebellum PCAs and PLS-DAs.
