# Supplementary material for: Effects of Alterations of Post-Mortem Delay and Other Tissue-Collection Variables on Metabolite Levels in Human and Rat Brain
Source: Metabolites. 2020 Oct 29;10(11):438. doi: 10.3390/metabo10110438 (PMC7694048; doi:10.3390/metabo10110438)
Supplement: Supplementary file 1 [file metabolites-10-00438-s001.zip › Supplementary Material A - Cohort Characteristics.docx]

Supplementary Material A

## Supplementary Table A1: Characteristics of individuals in the Manchester cohort

| Code | Gender | Age at death | Clinical diagnosis | Braak stage (AD) | APOE  Status | Post-Mortem Delay (hours) | Whole-brain weight (g) | CERAD | Thal | SVD | CAA | Brain pH | Cause of Death |
| --- | --- | --- | --- | --- | --- | --- | --- | --- | --- | --- | --- | --- | --- |
| AD1 | M | 88 | Alzheimer's Disease | V | 3/4 | 75 | 1027 | B | NA | Moderate | None | NA | NA |
| AD2 | M | 69 | Alzheimer's disease | VI | 3/3 | 96 | 1160† | C | NA | Mild | Moderate | NA | NA |
| AD3 | M | 65 | Alzheimer's disease | VI | 3/3 | *72** | NA | C | NA | None | Moderate | NA | NA |
| AD4 | F | 61 | Alzheimer's disease | VI | 3/4 | 130 | 1120 | C | NA | Mild | Mild | NA | NA |
| AD5 | F | 89 | Alzheimer's disease | V/VI | 3/3 | 72 | NA | C | 4 | Mild | Mild | NA | NA |
| AD6 | M | 76 | Alzheimer's disease | V/VI | 3/4 | *96** | 1359 | C | NA | None | Mild | NA | NA |
| AD7 | M | 83 | Alzheimer's disease | IV/V | 3/3 | 96 | 1046† | C | 3 | Mild | Mild/none | NA | NA |
| AD8 | F | 88 | Alzheimer's disease | VI | 3/3 | 72 | 900† | C | NA | Mild | Mild/none | NA | NA |
| AD9 | M | 87 | Alzheimer's disease | V | 3/4 | 49 | 1066 | B | NA | Severe | Severe | NA | NA |
| C1 | F | 92 | No dementia or brain disease | II | 3/4 | 37 | 1080† | A | 1 | Mild | None | NA | Myocardial infarction |
| C2 | F | 87 | No dementia or brain disease | I/II | 3/3 | 39 | 1160† | 0 | 0 | Moderate/severe | None | NA | Anteroseptal myocardial infarction; coronary atherosclerosis; left ventricular hypertrophy |
| C3 | M | 89 | No dementia or brain disease | II | 3/3 | 27 | 1400† | 0 | 0 | None | None | NA | Multi-organ failure, septicaemia, cellulitis, heart failure |
| C4 | M | 95 | No dementia or brain disease | I/II | 3/3 | 12 | 1200† | 0 | 0 | Mild | None | NA | Prostatic carcinoma |
| C5 | F | 87 | No dementia or brain disease | 0/I | 3/4 | 24 | 1152 | A | 1 | Mild/moderate | Moderate | NA | Cardiac failure, COPD, renal impairment, osteoarthritis |
| C6 | M | 84 | No dementia or brain disease | I | 3/3 | 69.5 | 1494 | A | 2 | Moderate | Mild | 5.84 | NA |
| C7 | F | 90 | No dementia or brain disease | 0/I | 3/3 | 39 | 1050 | 0 | 0 | None | None | NA | Frailty of old age |
| C8 | F | 82 | No dementia or brain disease | 0/I | 3/3 | 61 | 1020† | A | 3 | Mild | Absent/mild | NA | Metastatic ovarian cancer |
| C9 | F | 94 | No dementia or brain disease | I/II | 3/3 | 70 | 1276 | A | 3 | Mild | Mild | 5.87 | Carcinoma of the appendix |

*Estimated PMD (provided by the Manchester Brain Bank); †brain weighed at point of fixation by brain bank. Abbreviation: NA, not available

## Supplementary Table A2: Characteristics of individuals in the Newcastle cohort

| Code | Gender | Age at death | Clinical diagnosis | Braak stage (AD) | APOE  Status | Post-Mortem Delay (hours) | Whole-brain weight (g) | CERAD | Thal | SVD | CAA | Brain pH | Cause of Death |
| --- | --- | --- | --- | --- | --- | --- | --- | --- | --- | --- | --- | --- | --- |
| AD10 | M | 81 | Alzheimer's Disease | VI | 3/4 | 41 | 1351 | C | 5 | NA | Moderate/severe | 7.08 | Chest infection |
| AD11 | M | 87 | Alzheimer's disease | VI | 3/4 | 21 | 1200 | C | 5 | NA | Moderate/severe | 6.23 | Pneumonia |
| AD12 | F | 80 | Alzheimer's disease | VI | 4/4 | 10 | 985 | C | 5 | NA | Moderate/severe | 5.35 | Bronchopneumonia & Alzheimer’s disease |
| AD13 | F | 95 | Alzheimer's disease | VI | 3/4 | 23 | 968 | B | 5 | NA | Moderate/severe | 6.21 | Stroke |
| AD14 | M | 84 | Alzheimer's disease | VI | 3/4 | 40 | 1166 | B | 5 | NA | Moderate/severe | 6.70 | NA |
| AD15 | M | 86 | Alzheimer's disease | VI | 2/4 | 9 | 1066 | C | 5 | NA | Moderate/severe | 6.47 | Bronchopneumonia & metastatic bladder cancer |
| AD16 | M | 90 | Alzheimer's disease | VI | 2/3 | 13 | 1303 | C | 5 | NA | Moderate/severe | 6.43 | Frailty old age & chest infection & Alzheimer’s disease |
| AD17 | M | 86 | Alzheimer's disease | VI | 3/3 | 40 | 1043 | C | 5 | NA | Moderate/severe | 6.34 | NA |
| AD18 | F | 70 | Alzheimer's disease | VI | 4/4 | 24 | 959 | C | 5 | NA | Moderate/severe | 6.46 | Alzheimer’s disease |
| C10 | M | 88 | No dementia or brain disease | I | 2/3 | 28 | 1400 | 0 | 1 | NA | Moderate/severe | 6.30 | Chronic lymphocytic leukaemia |
| C11 | M | 80 | No dementia or brain disease | II | 3/3 | 16 | 1406 | 0 | 0 | NA | Moderate/Severe | 6.36 | Prostate cancer |
| C12 | M | 88 | No dementia or brain disease | II | 3/3 | 26 | 1362 | 0 | 3 | NA | NA | 6.10 | Chronic COPD |
| C13 | F | 81 | No dementia or brain disease | I | 3/3 | 40 | NA | NA | NA | NA | NA | NA | NA |

0

| C14 | M | 94 | No dementia or brain disease | III | 3/4 | 25 | 1175 | 0 | 1 | NA | Moderate/severe | 6.66 | Bronchopneumonia |
| --- | --- | --- | --- | --- | --- | --- | --- | --- | --- | --- | --- | --- | --- |
| C15 | F | 91 | No dementia or brain disease | II | 3/3 | 14 | 1238 | 0 | 0 | NA | NA | 6.55 | Aspiration pneumonia & severe inoperable pharyngeal pouch |
| C16 | M | 76 | No dementia or brain disease | II | 3/4 | 34 | 1363 | 0 | 2 | NA | NA | 6.52 | Pneumonic exacerbation of COPD |
| C17 | F | 81 | No dementia or brain disease | I | NA | 19 | 1064 | 0 | 3 | NA | Mild | 6.09 | Metastatic liver cancer |
| C18 | M | 92 | No dementia or brain disease | II | 2/3 | 9 | 1219 | 0 | 3 | NA | Moderate/severe | 6.28 | Uro-sepsis & metastatic prostate cancer, liver and adrenal metastasis & Chronic kidney disease |

Abbreviation: NA = Not available

## Supplementary Table A3: Characteristics of individuals in the Auckland cohort

| Code | Gender | Age at death | Clinical diagnosis | Braak stage (AD) | APOE  Status | Post-Mortem Delay (hours) | Whole-brain weight (g) | CERAD | Thal | SVD | CAA | Brain pH | Cause of Death |
| --- | --- | --- | --- | --- | --- | --- | --- | --- | --- | --- | --- | --- | --- |
| AD19 | M | 60 | Alzheimer's Disease | VI | NA | 7.0 | 1020 | NA | NA | NA | NA | 7.0 | Alzheimer’s disease |
| AD20 | F | 62 | Alzheimer's disease | VI | NA | 6.0 | 831 | NA | NA | NA | NA | 6.0 | Alzheimer’s disease |
| AD21 | F | 63 | Alzheimer's disease | VI | NA | 7.0 | 1080 | NA | NA | NA | NA | 7.0 | Bronchopneumonia |
| AD22 | F | 70 | Alzheimer's disease | V | NA | 7.0 | 1044 | NA | NA | NA | NA | 7.0 | Lung cancer |
| AD23 | M | 73 | Alzheimer's disease | IV | NA | 4.0 | 1287 | NA | NA | NA | NA | 4.0 | GI haemorrhage |
| AD24 | F | 74 | Alzheimer's disease | V | NA | 8.5 | 1062 | NA | NA | NA | NA | 8.5 | Metastatic cancer |
| AD25 | M | 74 | Alzheimer's disease | VI | NA | 12.0 | 1355 | NA | NA | NA | NA | 12.0 | Pseudomonas bacteraemia |
| AD26 | M | 77 | Alzheimer's disease | VI | NA | 4.5 | 1180 | NA | NA | NA | NA | 4.5 | Myocardial infarction |
| AD27 | M | 80 | Alzheimer's disease | V | NA | 5.5 | 1039 | NA | NA | NA | NA | 5.5 | Bronchopneumonia/ pulmonary oedema |
| C19 | M | 61 | No dementia or brain disease | 0 | NA | 7.0 | 1258 | NA | NA | NA | NA | 7.0 | Ischaemic heart disease |
| C20 | F | 64 | No dementia or brain disease | 0 | NA | 5.5 | 1260 | NA | NA | NA | NA | 5.5 | Pulmonary embolism |
| C21 | F | 63 | No dementia or brain disease | 0 | NA | 12.0 | 1280 | NA | NA | NA | NA | 12.0 | Ruptured aorta |
| C22 | F | 72 | No dementia or brain disease | 0 | NA | 9.0 | 1230 | NA | NA | NA | NA | 9.0 | Emphysema |
| C23 | M | 66 | No dementia or brain disease | 0 | NA | 9.0 | 1461 | NA | NA | NA | NA | 9.0 | Ischaemic heart disease |
| C24 | F | 76 | No dementia or brain disease | II | NA | 12.0 | 1094 | NA | NA | NA | NA | 12.0 | Metastatic carcinoma |
| C25 | M | 73 | No dementia or brain disease | 0 | NA | 13.0 | 1315 | NA | NA | NA | NA | 13.0 | Ischaemic heart disease |
| C26 | M | 78 | No dementia or brain disease | 0 | NA | 7.5 | 1260 | NA | NA | NA | NA | 7.5 | Ruptured aortic aneurysm |
| C27 | M | 78 | No dementia or brain disease | 0 | NA | 12.0 | 1416 | NA | NA | NA | NA | 12.0 | Ruptured MI |

Abbreviation: NA = Not available.

## Supplementary Figure A1: CERAD Score vs tau Braak Stage

Scatter plot shows individual CERAD/Braak scores with medians. A Spearman's rank-order correlation was run to determine the relationship between CERAD score and tau Braak Stage. There was a strong, positive correlation which was statistically significant (r^2^ = 0.8148; p < 0.001).
