# Supplementary material for: Effects of Alterations of Post-Mortem Delay and Other Tissue-Collection Variables on Metabolite Levels in Human and Rat Brain
Source: Metabolites. 2020 Oct 29;10(11):438. doi: 10.3390/metabo10110438 (PMC7694048; doi:10.3390/metabo10110438)

Supplementary Material C – Human Cohort PCA & PLS-DA Plots

Contents

[Supplementary Figure C1. Manchester Cohort PCAs and PLS-DAs 2](#_Toc54866367)

[A) PCA Plot without QCs and QLs (Unlabelled) 2](#_Toc54866368)

[B) PCA Plot without QCs and QLs (Labelled) 2](#_Toc54866369)

[C) PCA Plot with QCs and QLs (Unlabelled) 3](#_Toc54866370)

[D) PCA Plot with QCs and QLs (Labelled) 3](#_Toc54866371)

[E) PLS-DA Plot without QCs and QLs (Unlabelled) 4](#_Toc54866372)

[F) PLS-DA Plot without QCs and QLs (Labelled) 4](#_Toc54866373)

[G) PLS-DA Plot with QCs and QLs (Labelled) 5](#_Toc54866374)

[H) PLS-DA Plot with QCs and QLs (Labelled) 5](#_Toc54866375)

[I) PCA Plot with Outliers Included (Labelled) 6](#_Toc54866376)

[Supplementary Figure C2. Newcastle Cohort PCAs and PLS-DAs 7](#_Toc54866377)

[A) PCA Plot without QCs and QLs (Unlabelled) 7](#_Toc54866378)

[B) PCA Plot without QCs and QLs (Labelled) 7](#_Toc54866379)

[C) PCA Plot with QCs and QLs (Unlabelled) 8](#_Toc54866380)

[D) PCA Plot with QCs and QLs (Labelled) 8](#_Toc54866381)

[E) PLS-DA Plot without QCs and QLs (Unlabelled) 9](#_Toc54866382)

[F) PLS-DA Plot without QCs and QLs (Labelled) 9](#_Toc54866383)

[G) PLS-DA Plot with QCs and QLs (Unlabelled) 10](#_Toc54866384)

[H) PLS-DA Plot with QCs and QLs (Labelled) 10](#_Toc54866385)

# Supplementary Figure C1. Manchester Cohort PCAs and PLS-DAs

## PCA Plot without QCs and QLs (Unlabelled)


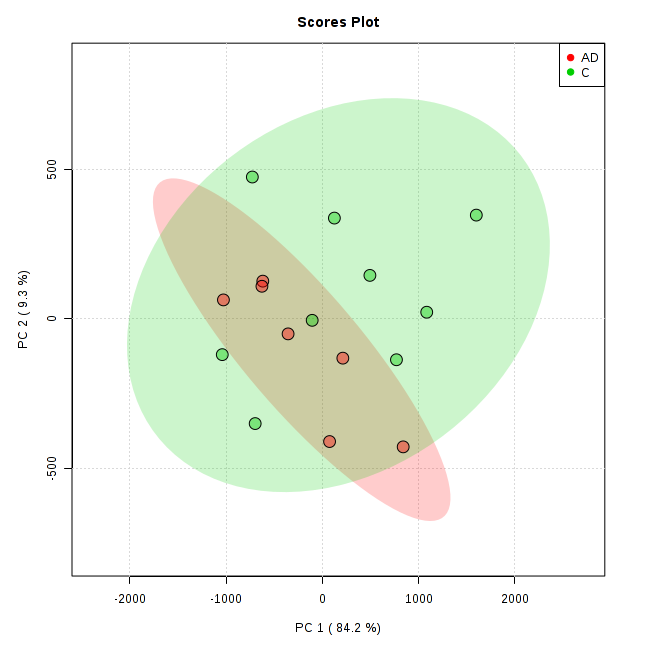


## PCA Plot without QCs and QLs (Labelled)


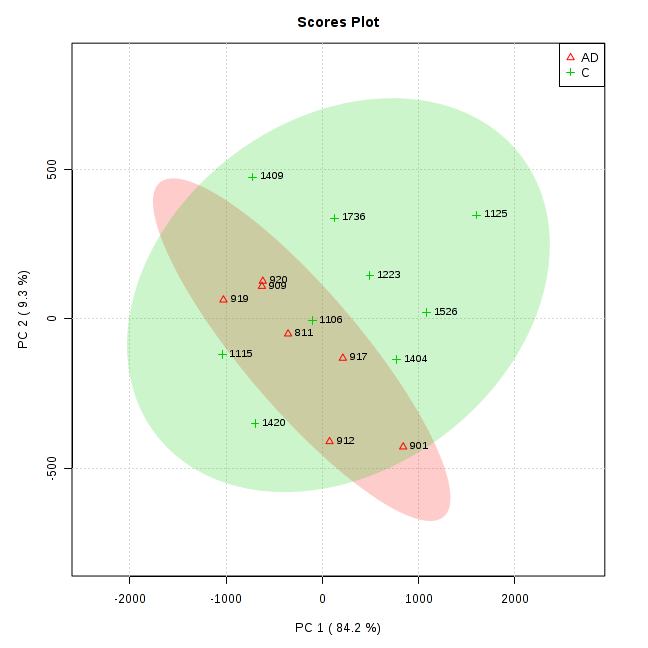


## PCA Plot with QCs and QLs (Unlabelled)


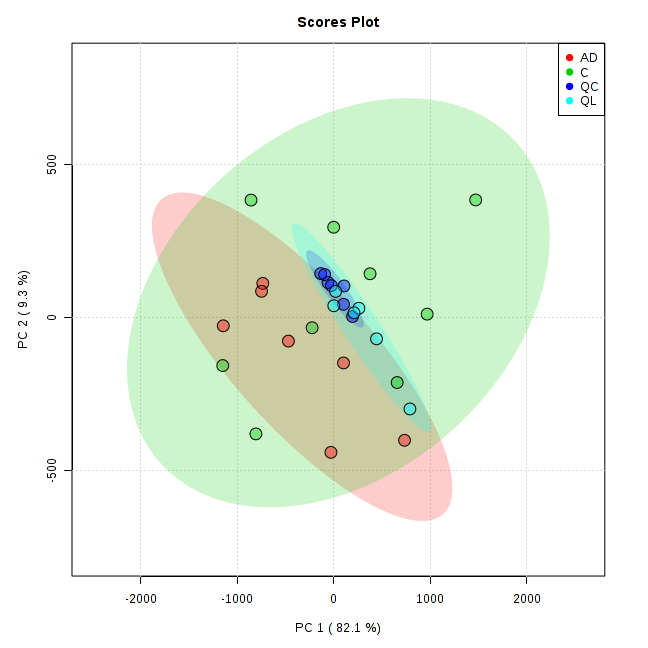


## PCA Plot with QCs and QLs (Labelled)


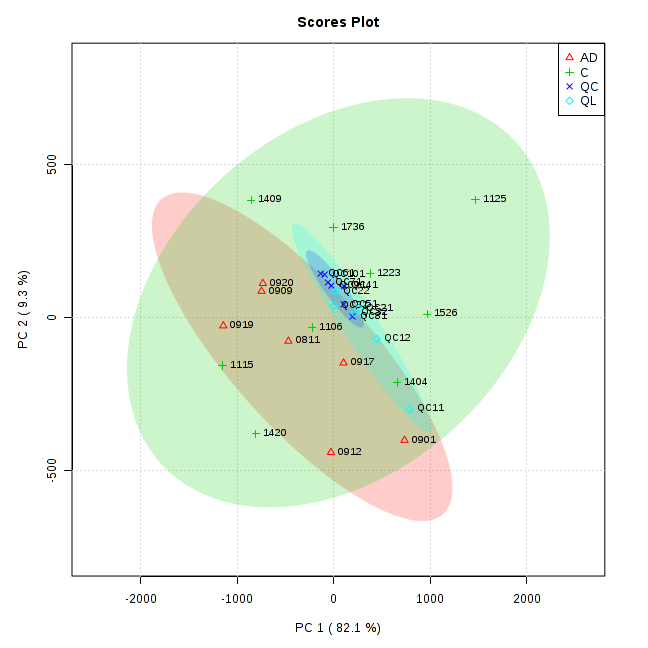


## PLS-DA Plot without QCs and QLs (Unlabelled)


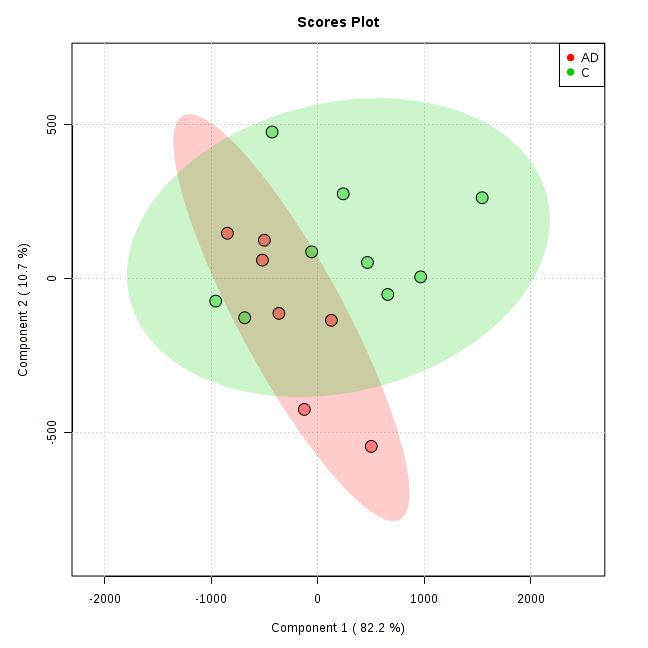


## PLS-DA Plot without QCs and QLs (Labelled)


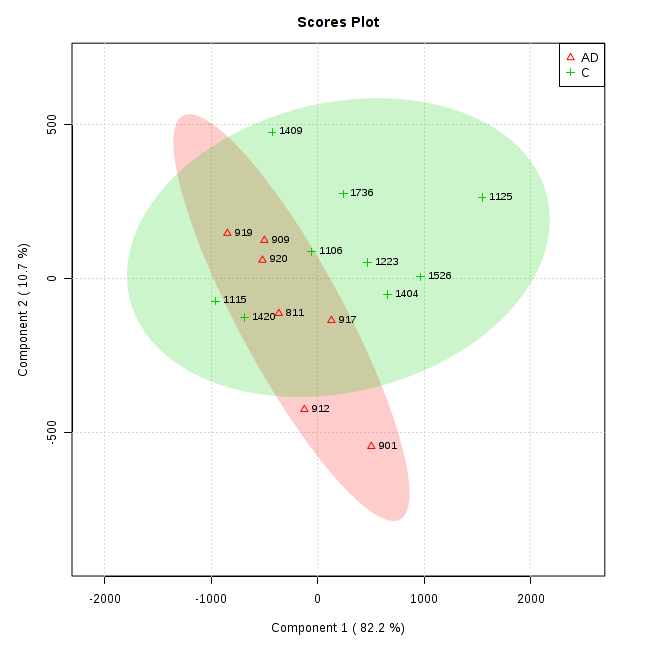


## PLS-DA Plot with QCs and QLs (Labelled)


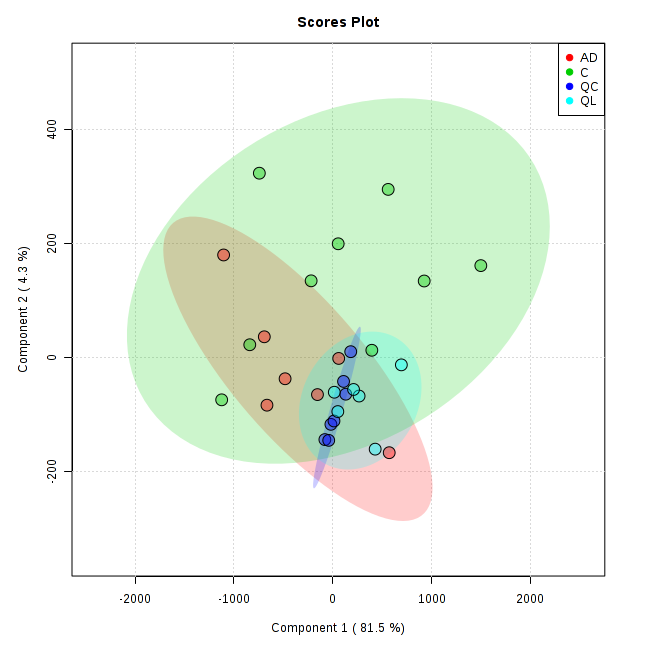


## PLS-DA Plot with QCs and QLs (Labelled)


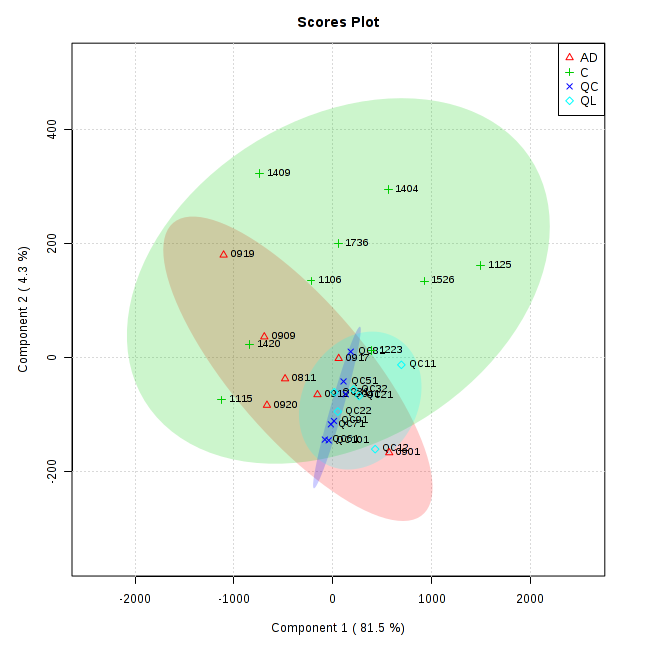


## PCA Plot with Outliers Included (Labelled)


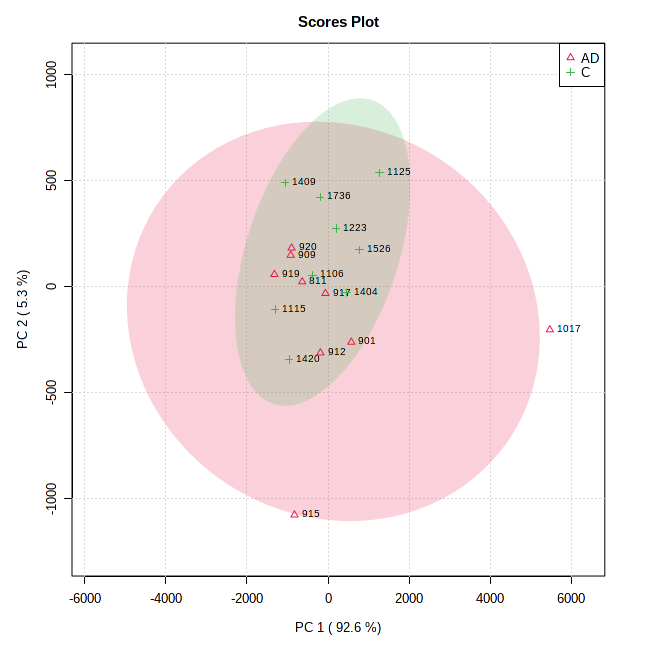


# Supplementary Figure C2. Newcastle Cohort PCAs and PLS-DAs

## PCA Plot without QCs and QLs (Unlabelled)


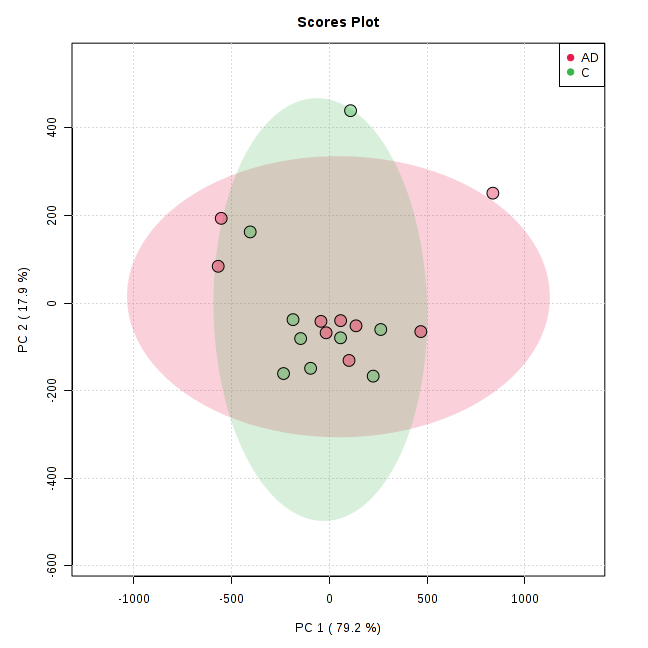


## PCA Plot without QCs and QLs (Labelled)


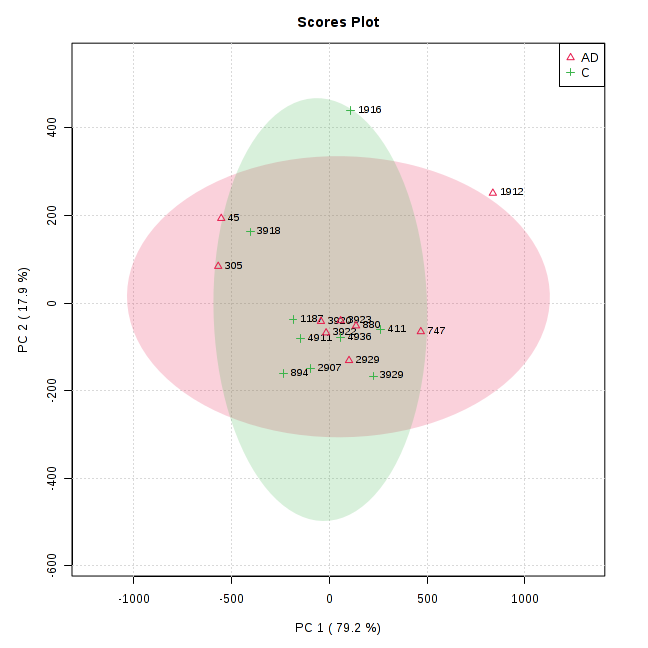


## PCA Plot with QCs and QLs (Unlabelled)


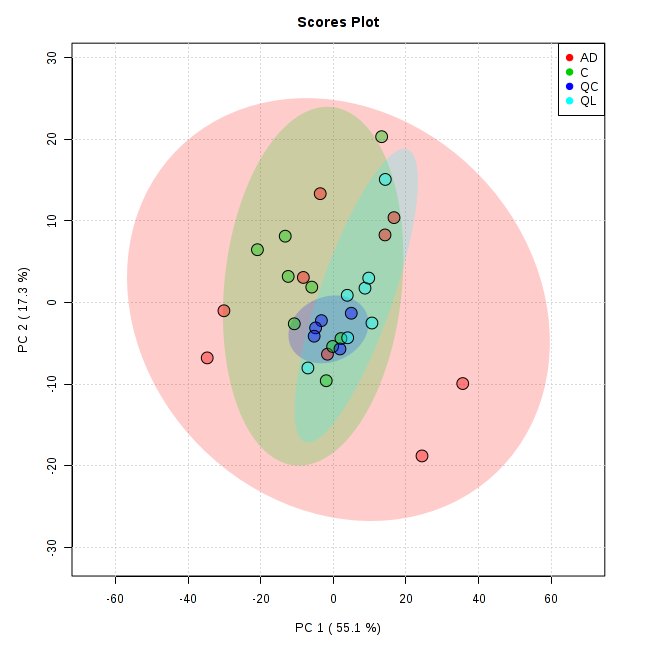


## PCA Plot with QCs and QLs (Labelled)


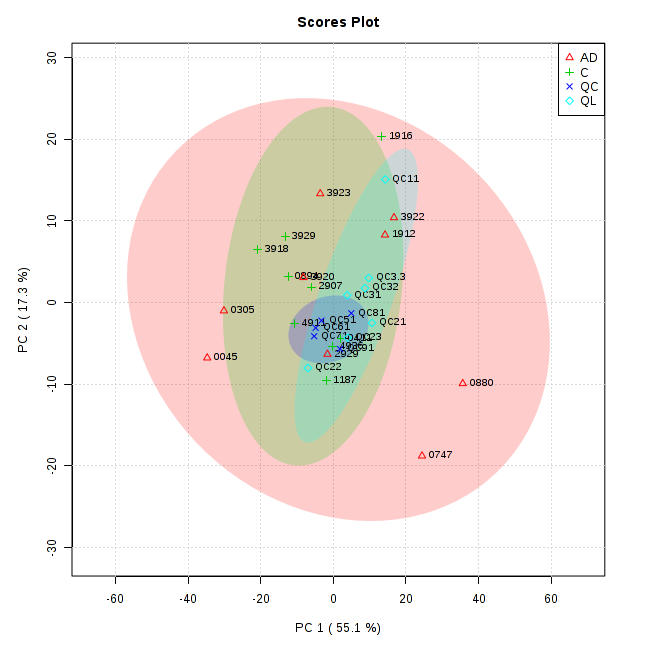


## PLS-DA Plot without QCs and QLs (Unlabelled)


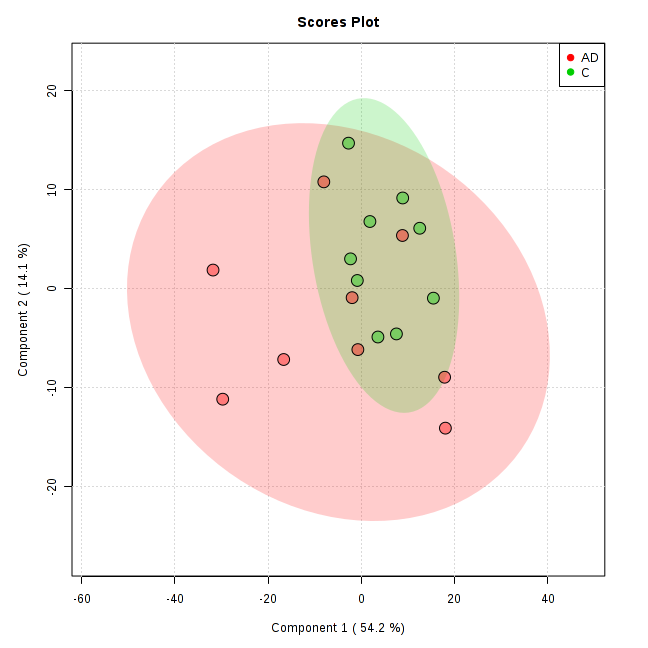


## PLS-DA Plot without QCs and QLs (Labelled)


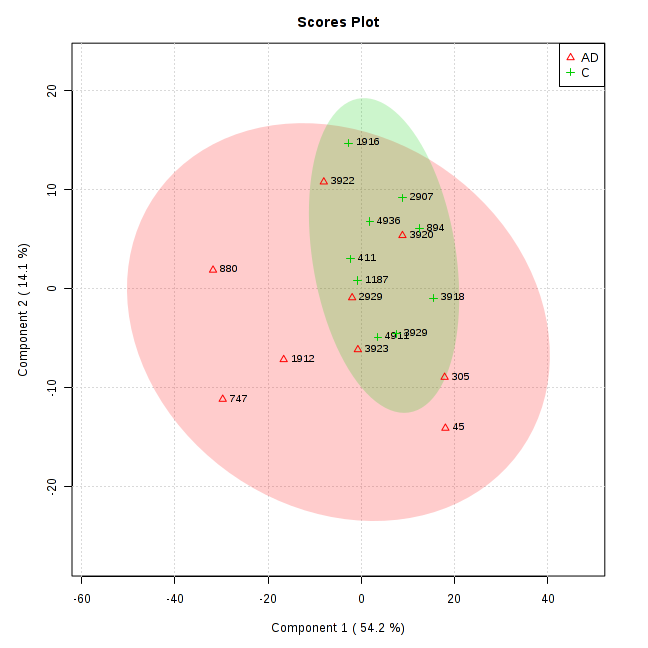


## PLS-DA Plot with QCs and QLs (Unlabelled)


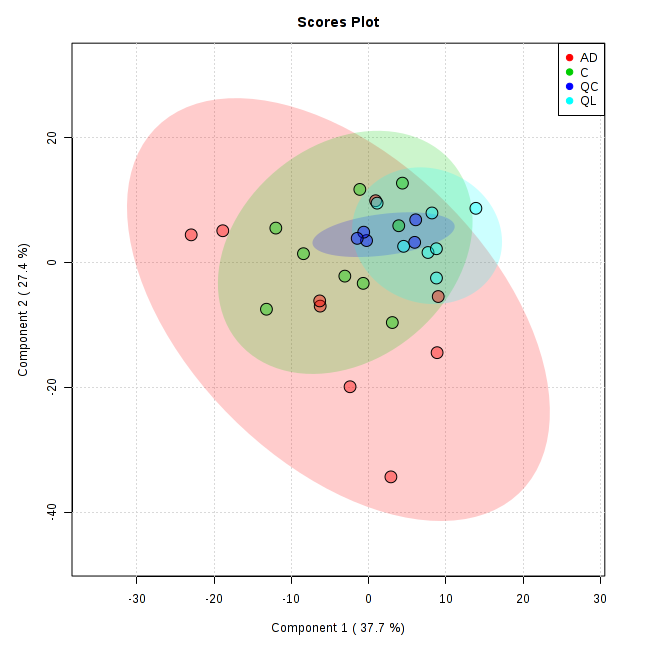


## PLS-DA Plot with QCs and QLs (Labelled)


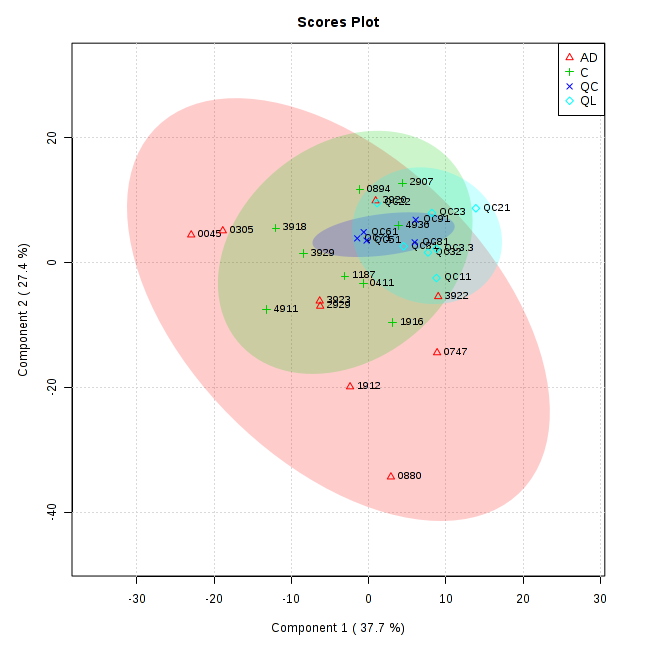

Supplement: Supplementary file 1 [file metabolites-10-00438-s001.zip › Supplementary Material C - Human Brain Plots.docx]
