# Supplementary material for: Effects of Alterations of Post-Mortem Delay and Other Tissue-Collection Variables on Metabolite Levels in Human and Rat Brain
Source: Metabolites. 2020 Oct 29;10(11):438. doi: 10.3390/metabo10110438 (PMC7694048; doi:10.3390/metabo10110438)

Supplementary Material E – Rat Brain PCA & PLS-DA Plots

Contents

[Supplementary Figure E1. Rat Cortex PCAs and PLS-DAs 2](#_Toc54866440)

[A) PCA Plot without QCs and QLs (Unlabelled) 2](#_Toc54866441)

[B) PCA Plot without QCs and QLs (Labelled) 2](#_Toc54866442)

[C) PCA Plot with QCs (Unlabelled) 3](#_Toc54866443)

[D) PCA Plot with QCs and QLs (Labelled) 3](#_Toc54866444)

[E) PLS-DA Plot without QCs and QLs (Unlabelled) 4](#_Toc54866445)

[F) PLS-DA Plot without QCs and QLs (Labelled) 4](#_Toc54866446)

[G) PLS-DA Plot with QCs and QLs (Unlabelled) 5](#_Toc54866447)

[H) PLS-DA Plot with QCs and QLs (Labelled) 5](#_Toc54866448)

[I) PCA Plot with Outliers Included (Labelled) 6](#_Toc54866449)

[Supplementary Figure E2. Rat Cerebellum PCAs and PLS-DAs 7](#_Toc54866450)

[A) PCA Plot without QCs and QLs (Unlabelled) 7](#_Toc54866451)

[B) PCA Plot without QCs and QLs (Labelled) 7](#_Toc54866452)

[C) PCA Plot with QCs and QLs (Unlabelled) 8](#_Toc54866453)

[D) PCA Plot with QCs and QLs (Labelled) 8](#_Toc54866454)

[E) PLS-DA Plot without QCs and QLs (Unlabelled) 9](#_Toc54866455)

[F) PLS-DA Plot without QCs and QLs (Labelled) 9](#_Toc54866456)

[G) PLS-DA Plot with QCs and QLs (Unlabelled) 10](#_Toc54866457)

[H) PLS-DA Plot with QCs and QLs (Labelled) 10](#_Toc54866458)

[I) PCA Plot with Outliers Included (Labelled) 11](#_Toc54866459)

# Supplementary Figure E1. Rat Cortex PCAs and PLS-DAs

## PCA Plot without QCs and QLs (Unlabelled)


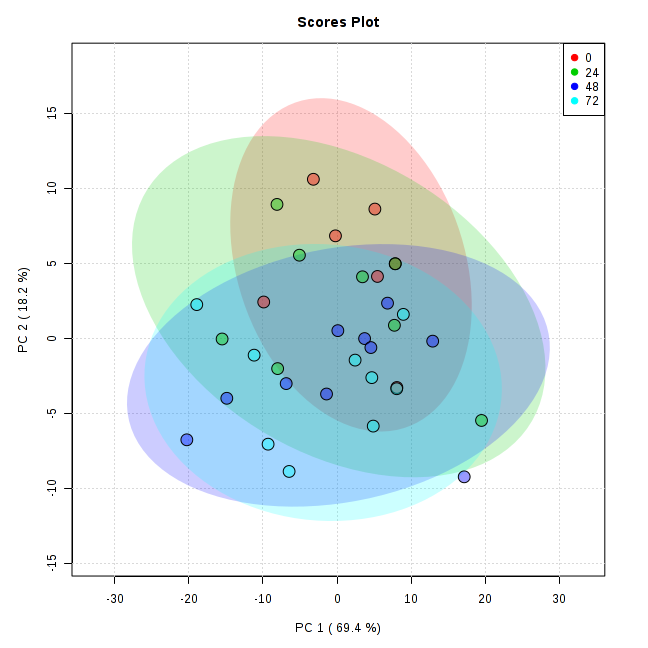


## PCA Plot without QCs and QLs (Labelled)


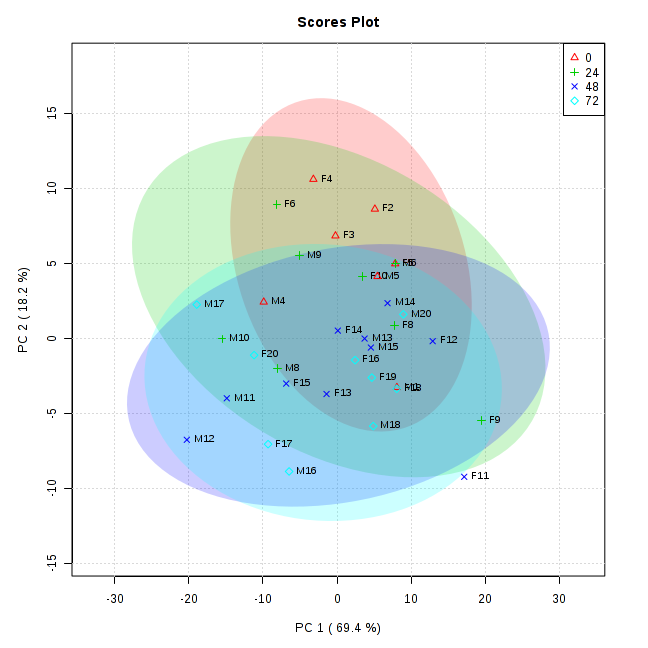


## PCA Plot with QCs (Unlabelled)


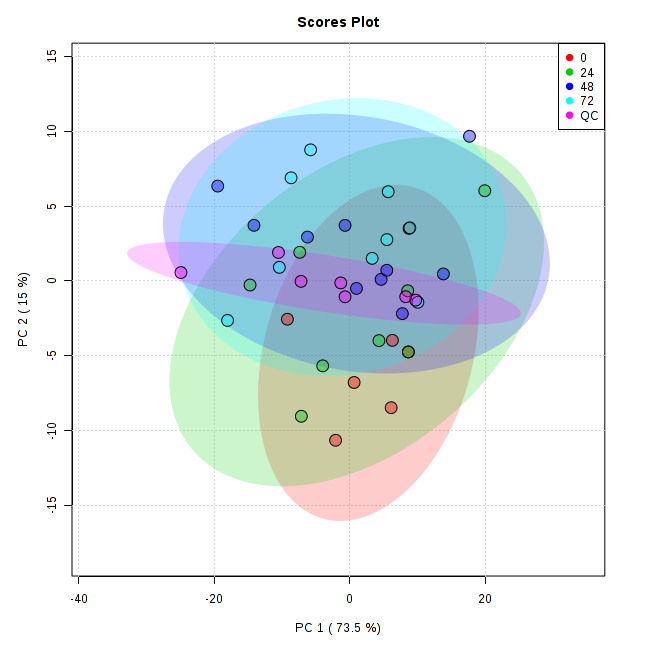


## PCA Plot with QCs and QLs (Labelled)


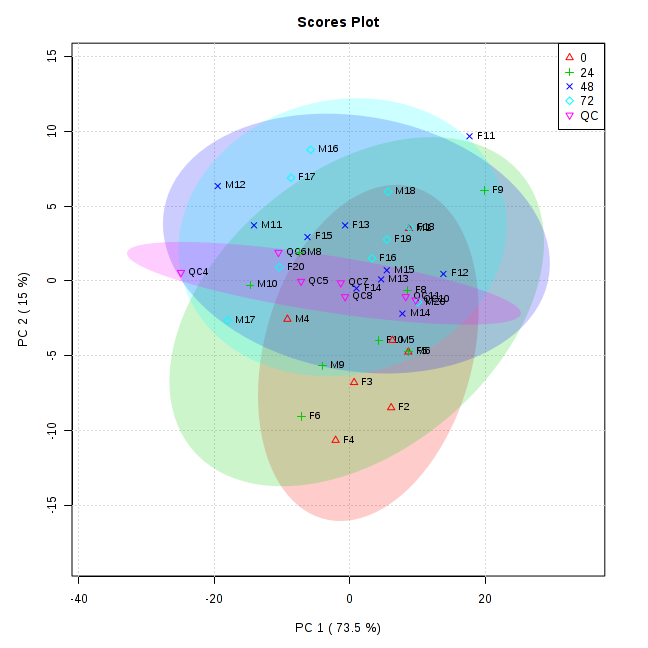


## PLS-DA Plot without QCs and QLs (Unlabelled)


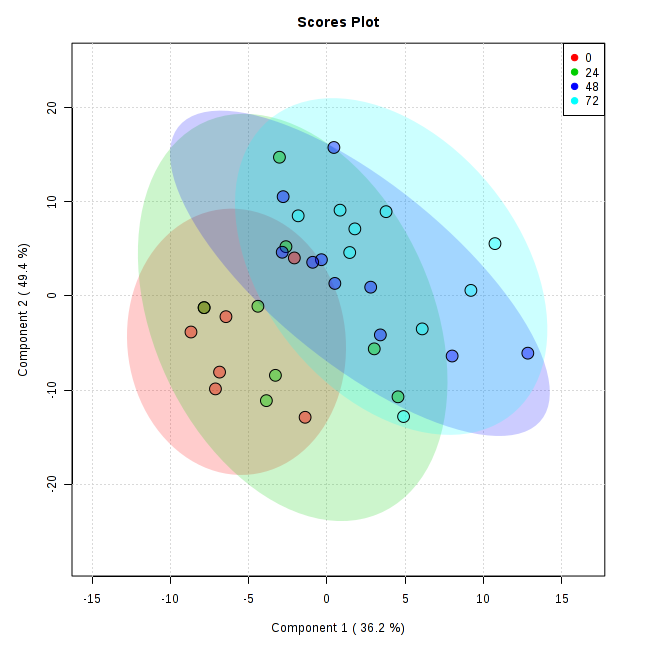


## PLS-DA Plot without QCs and QLs (Labelled)


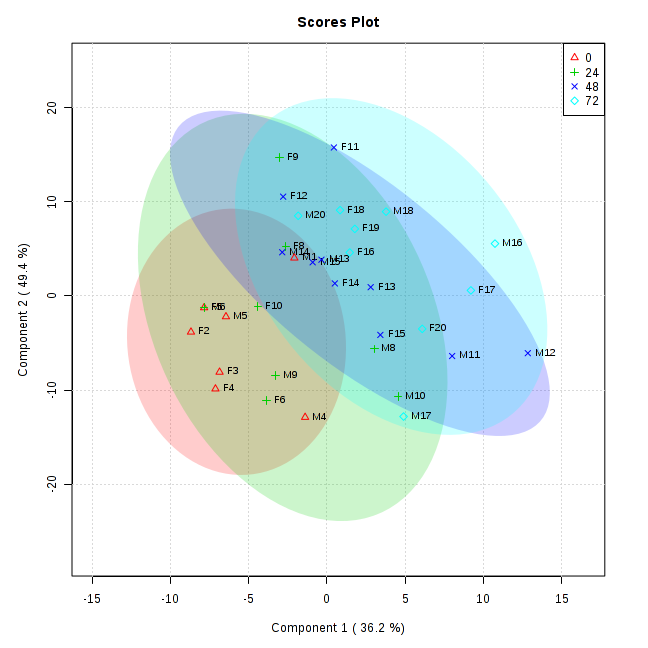


## PLS-DA Plot with QCs and QLs (Unlabelled)


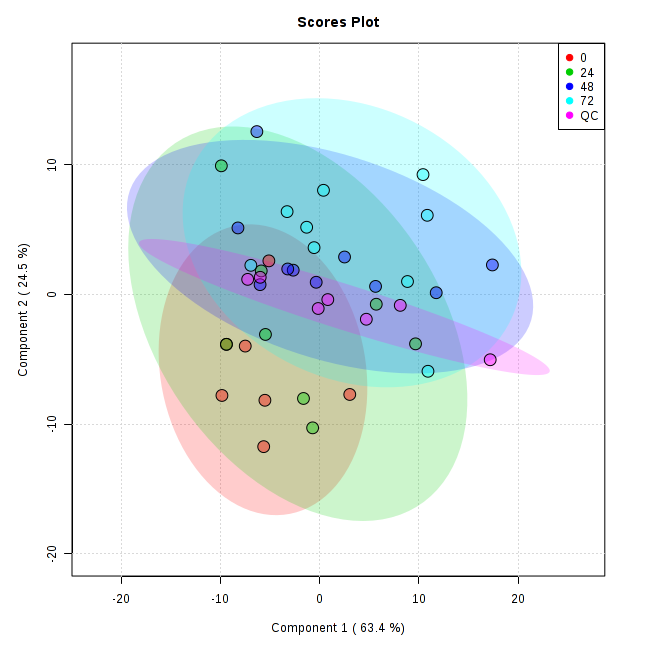


## PLS-DA Plot with QCs and QLs (Labelled)


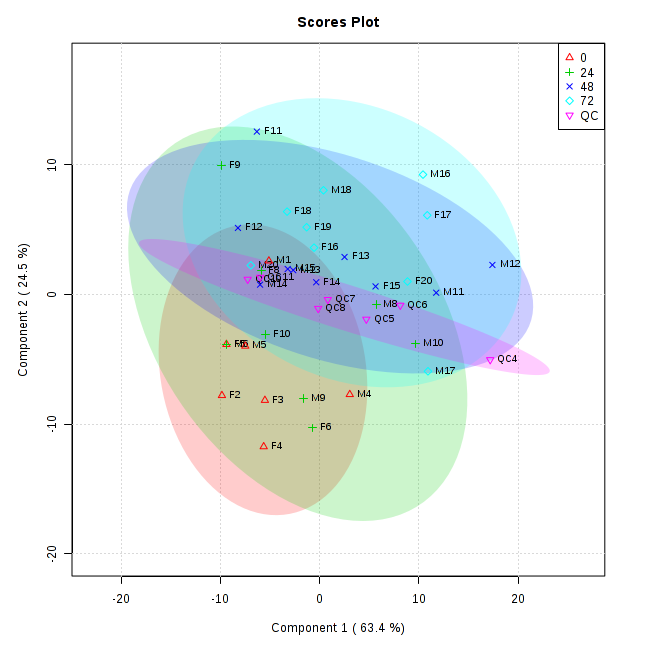


## PCA Plot with Outliers Included (Labelled)


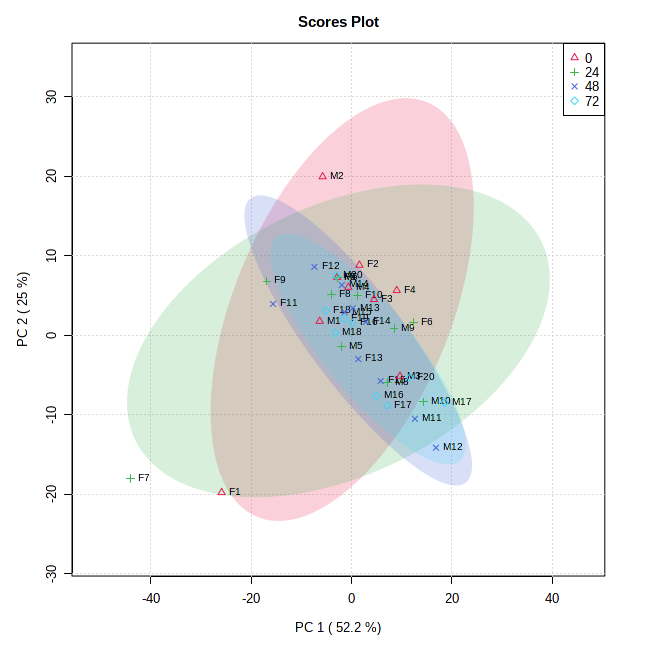


# Supplementary Figure E2. Rat Cerebellum PCAs and PLS-DAs

## PCA Plot without QCs and QLs (Unlabelled)


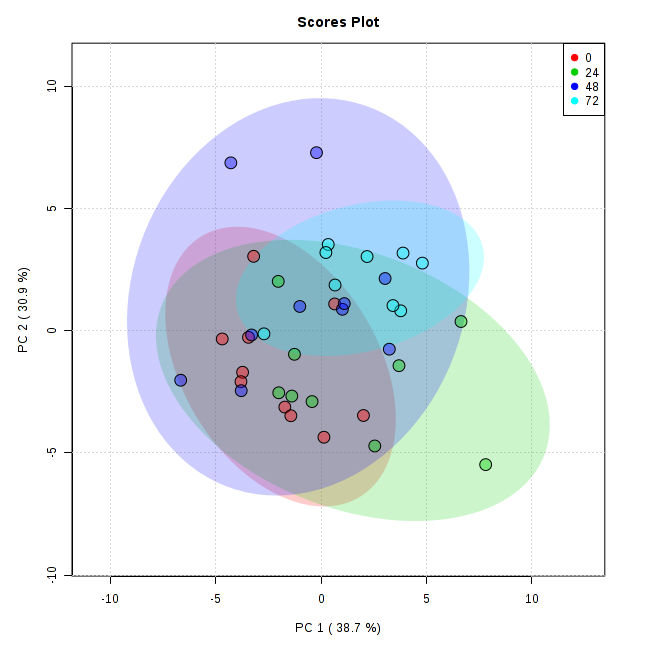


## PCA Plot without QCs and QLs (Labelled)


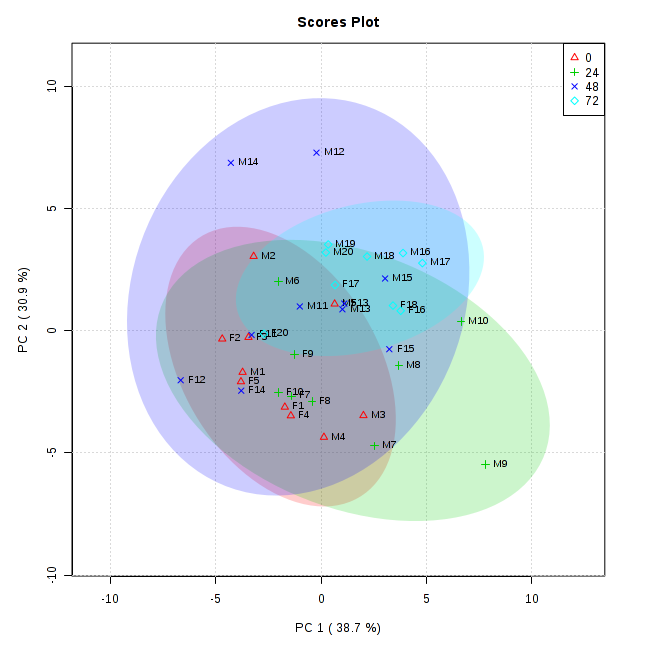


## PCA Plot with QCs and QLs (Unlabelled)


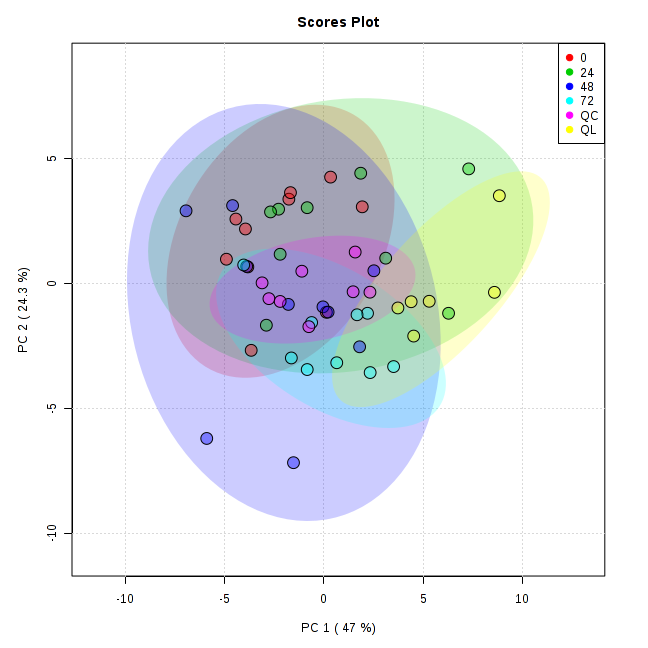


## PCA Plot with QCs and QLs (Labelled)


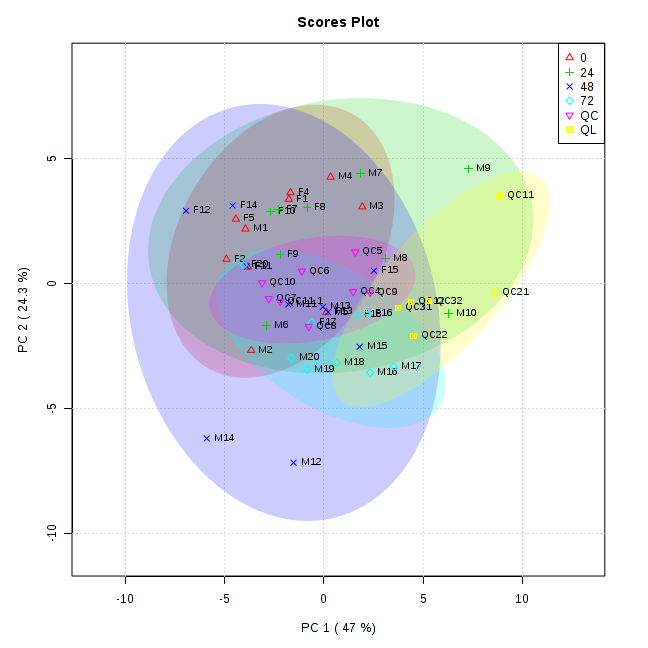


## PLS-DA Plot without QCs and QLs (Unlabelled)


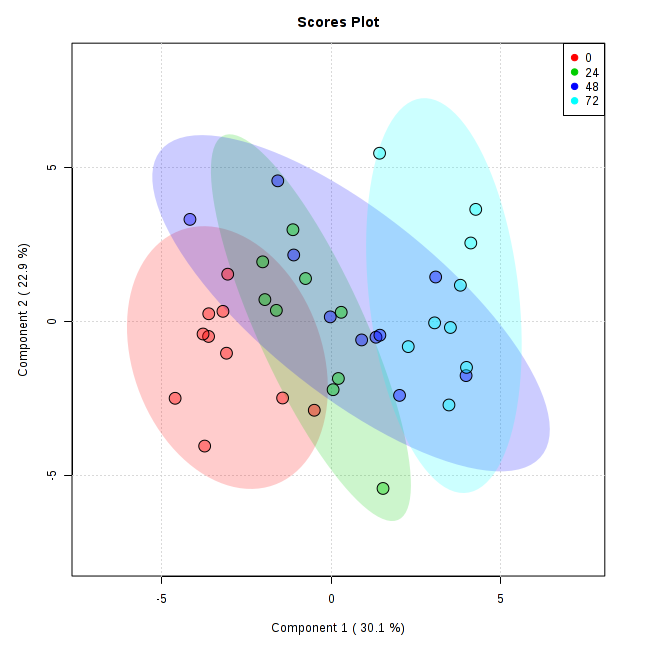


## PLS-DA Plot without QCs and QLs (Labelled)


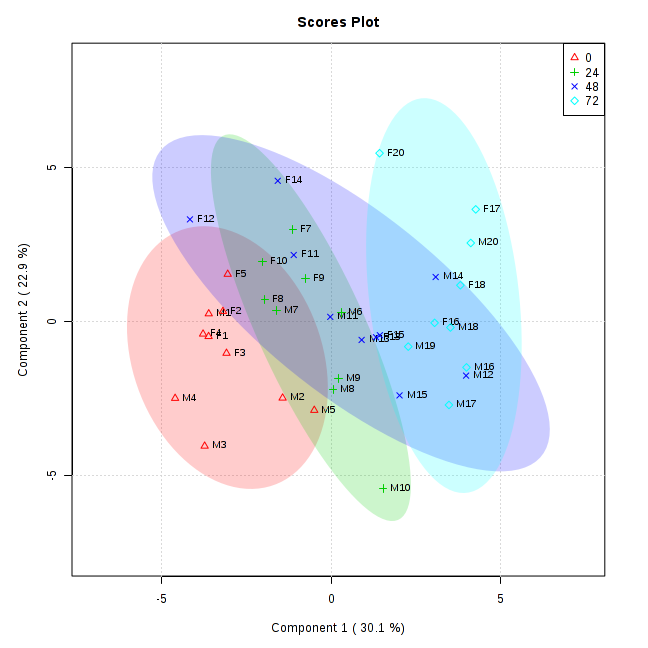


## PLS-DA Plot with QCs and QLs (Unlabelled)


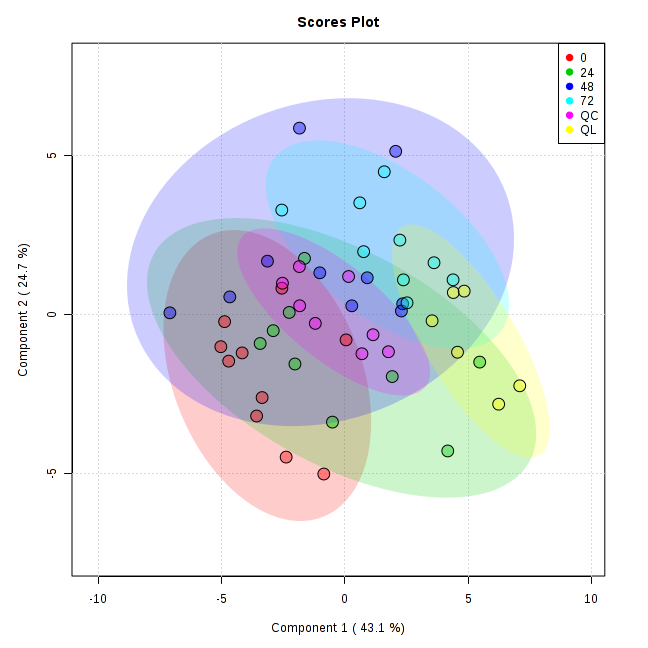


## PLS-DA Plot with QCs and QLs (Labelled)


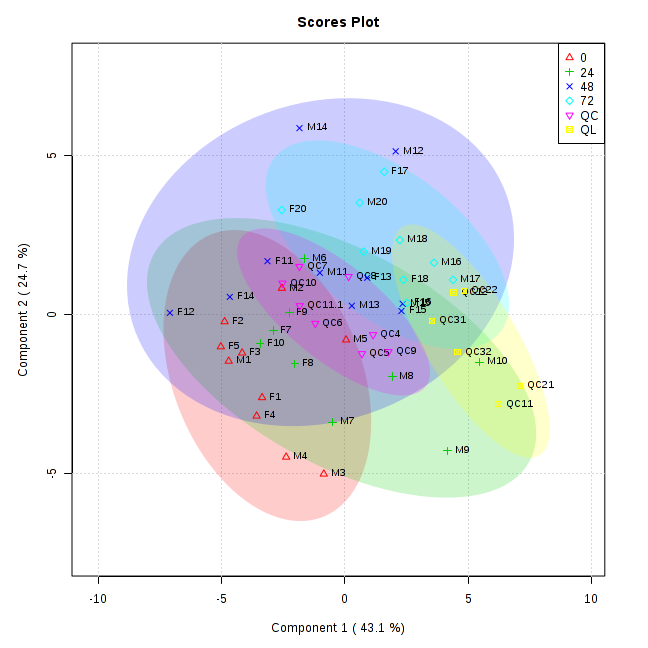


## PCA Plot with Outliers Included (Labelled)


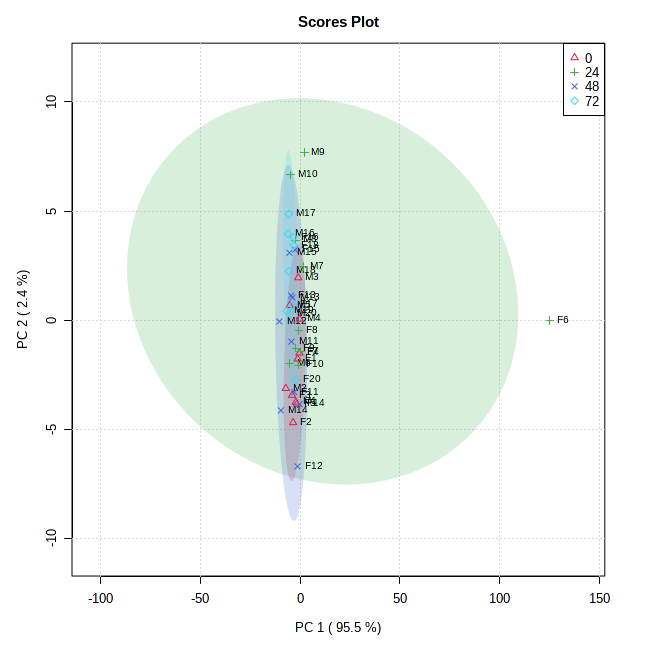

Supplement: Supplementary file 1 [file metabolites-10-00438-s001.zip › Supplementary Material E - Rat Brain Plots.docx]
